# Hyperactive mTORC1 in striatum dysregulates dopamine receptor expression and odor preference behavior

**DOI:** 10.3389/fnins.2024.1461178

**Published:** 2024-08-30

**Authors:** Lin Chen, Ryo Saito, Shoko Noda-Narita, Hidetoshi Kassai, Atsu Aiba

**Affiliations:** ^1^Laboratory of Animal Resources, Center for Disease Biology and Integrated Medicine, Graduate School of Medicine, The University of Tokyo, Tokyo, Japan; ^2^Department of Biological Sciences, Graduate School of Science, The University of Tokyo, Tokyo, Japan; ^3^Central Animal Division, National Cancer Center Research Institute, Tokyo, Japan

**Keywords:** mTOR, inhibitory neuron, dopamine receptor, medium spiny neuron, olfactory tubercle, olfactory behavior

## Abstract

Mechanistic target of rapamycin (mTOR) plays an important role in brain development and synaptic plasticity. Dysregulation of the mTOR pathway is observed in various human central nervous system diseases, including tuberous sclerosis complex, autism spectrum disorder (ASD), and neurodegenerative diseases, including Parkinson’s disease and Huntington’s disease. Numerous studies focused on the effects of hyperactivation of mTOR on cortical excitatory neurons, while only a few studies focused on inhibitory neurons. Here we generated transgenic mice in which mTORC1 signaling is hyperactivated in inhibitory neurons in the striatum, while cortical neurons left unaffected. The hyperactivation of mTORC1 signaling increased GABAergic inhibitory neurons in the striatum. The transgenic mice exhibited the upregulation of dopamine receptor D1 and the downregulation of dopamine receptor D2 in medium spiny neurons in the ventral striatum. Finally, the transgenic mice demonstrated impaired motor learning and dysregulated olfactory preference behavior, though the basic function of olfaction was preserved. These findings reveal that the mTORC1 signaling pathway plays an essential role in the development and function of the striatal inhibitory neurons and suggest the critical involvement of the mTORC1 pathway in the locomotor abnormalities in neurodegenerative diseases and the sensory defects in ASD.

## 1 Introduction

mTOR is an evolutionarily conserved Ser/Thr protein kinase. It interacts with multiple proteins and forms two distinct protein complexes, termed mTORC1 and mTORC2 (Albert and Hall, 2015). mTORC1 is a master growth regulator involved in various cellular processes, including cell growth, proliferation, and survival. Several environmental cues can stimulate the mTORC1 pathway, including energy, stress, amino acids and growth factors (Foster and Fingar, 2010). mTORC1 promotes protein synthesis by phosphorylation of p70 S6 kinase (S6K1 and S6K2) and eukaryotic initiation factor 4-binding protein (4EBP1 and 4EBP2). As mTORC1 is an important regulator of many cellular processes, dysregulation of mTORC1 signaling contributes to a variety of human diseases, including cancer and metabolic diseases (Laplante and Sabatini, 2012). mTORC1 also plays an important role in development and synaptic plasticity in the central nervous systems (CNS) (Jaworski and Sheng, 2006). Dysregulation of mTORC1 signaling is observed in several human CNS disorders, such as tuberous sclerosis complex (TSC), fragile X syndrome, and neurodegenerative diseases (Orlova and Crino, 2010; Busquets-Garcia et al., 2013; Sato et al., 2012; Bové et al., 2011). Animal models of these mTORC1-related diseases are conventionally established by loss of *Tsc1, Tsc2* or *Pten*, but our group previously generated transgenic (Tg) mice expressing a gain-of-function mutant of mTOR (Kassai et al., 2014), which directly and constitutively activates the mTORC1 signaling. Excitatory neuron-specific activation of mTORC1 signaling in the forebrain leads to lethal seizure and recapitulates TSC and neurodegeneration. However, little is known about the mTORC1 dysregulation in inhibitory neurons.

Medium spiny neurons (MSNs) are a specific type of GABAergic inhibitory neurons which represents 90% of the neurons in human dorsal striatum (neostriatum, caudate putamen). The dorsal striatum controls the motor and reward systems. MSNs have two characteristic subtypes, called dopamine receptor D1-expressing (D1-type) and dopamine receptor D2-expressing (D2-type) MSNs (Yager et al., 2015). D1- and D2-type MSNs are mainly involved in striatal direct and indirect pathways, respectively. The dyscoordination of D1-type and D2-type MSNs is observed in neurodegenerative diseases, including Parkinson’s disease and Huntington’s disease. In recent studies, dysregulation of mTORC1 signaling is shown to contribute to the dysfunction of MSNs in these neurodegenerative diseases (Santini et al., 2009; Pryor et al., 2014). Further, the involvement of the dorsal striatal activation of mTORC1 signaling in the repetitive behaviors in ASD was recently shown using *Tsc1*-KO mouse model (Benthall et al., 2021). However, it is unclear how activation of the mTORC1 pathway directly affects D1- or D2-type MSN population of the striatum and how the discoordination of MSNs affects the downstream neuronal circuits. Moreover, the ventral striatum, which includes nucleus accumbens and olfactory tubercle (OT), also processes the motivation, reward, and fear (Ikemoto, 2007). Though the ventral striatum contains D1- and D2-type MSNs and is dysregulated in psychiatric disorders, almost no studies have analyzed the relationship between mTORC1 signaling and neuronal dysfunction in the ventral striatum.

There are a few animal models of mTOR-related diseases focusing on the inhibitory neurons. GABAergic interneuron-specific *Tsc1*-KO mice using *Dlx5/6*-Cre mice showed impaired growth and decreased survival with seizure susceptibility (Fu et al., 2012). The conditional *Tsc1*-KO mice had enlarged soma size and impaired migration of the cortical interneurons. Somatostatin (SST)-specific and parvalbumin (PV)-specific conditional KO of *Tsc1* also demonstrated the disrupted morphological and functional development of cortical interneurons and deficits in social behavior (Malik et al., 2019; Amegandjin et al., 2021). These studies suggest that hyperactive mTORC1 signaling in cortical interneurons may be responsible for human *TSC* patient-like spontaneous seizures and ASD-related social abnormalities. ASD-related behavioral phenotypes are also observed in an animal model generated by deleting *Pten* gene in SST-specific and PV-specific interneurons (Shin et al., 2021). The conditional *Pten*-KO mice exhibited impaired motor learning and social deficits. However, most of those animal models with hyperactivation of mTORC1 pathway in inhibitory neurons showed abnormalities according to the dysfunction of cortical interneurons. Only a few studies have focused on the mTORC1 function in the striatum and almost no animal models were generated to figure out the mTORC1 contribution to the development and function in striatal MSNs.

In the present study, to address mTORC1-specific contribution in the function of dorsal and ventral striatum, we generated Tg mice in which mTORC1 signaling is constitutively activated in the dorsal and ventral striatal MSNs. Hyperactivation of mTORC1 increased the cell number and the cell size of GABAergic inhibitory neurons in the striatum, and the Tg mice exhibited impaired motor learning. Furthermore, the hyperactivation of mTORC1 increased D1-type MSNs, while decreasing D2-type MSNs in the OT, and olfactory preference behavior was disrupted in the Tg mice. Taken together, the mTORC1 signaling plays a critical role in the proliferation and function of the striatal MSNs.

## 2 Materials and methods

### 2.1 Mice

All animal experiments were approved by the Institutional Animal Care and Use Committee of the University of Tokyo (Permit No. P21-038 and A2023M004) and were conducted in accordance with the guidelines of the University of Tokyo. All mice were housed under specific pathogen-free conditions with a temperature of 23 ± 1°C, humidity of 50 ± 10%, a 12-h light/12-h dark cycle (light: 8:00–20:00/ dark: 20:00–8:00), in cages with food and water available *ad libitum*.

Dlx1-CreER^T2^ mice (JAX stock #014551) (Taniguchi et al., 2011) and ROSA26R-lacZ mice (JAX stock # 003474) (Soriano, 1999) were provided by Dr. Hiroki Kurihara. CAG-mTOR Tg mice were generated as described previously (Kassai et al., 2014). In CAG-mTOR Tg mice, active mTOR^SL1 + IT^ can be conditionally expressed upon excision of the *loxP*-flanked *neo* gene in response to Cre-*loxP* recombination. CAG-mTOR Tg mice were crossed with Dlx1-CreER^T2^ mice to obtain Dlx1-mTOR Tg mice (*Dlx1-CreER^T2^/+; CAG-mTOR^SL1 + IT^/+*) and the control mice (*CAG-mTOR^SL1 + IT^/+*). Genetic background of the Tg mice used in this study was a hybrid of C57BL/6 and ICR. For induction of Cre recombinase activity, tamoxifen (T5648, SIGMA) was dissolved in sesame oil and administered to the pregnant mice by oral gavage at a dose of 0.1 mg/g body weight at 12.5 day postcoitum (dpc), and the pups were used for the histological and behavioral experiments. For behavioral tests, 8- to 14-week-old mice were used, and mice were placed in the testing room for at least 1 h to acclimate to the experimental environments. In open field test, Y-maze test, elevated plus maze test and rota-rod test, male and female mice were analyzed separately. In olfactory habituation and dishabituation test, buried food-seeking test and three-chamber odor preference test, both male and female mice were included in the same analyses. For histological analysis, 14-week-old mice were used.

### 2.2 *β*- galactosidase staining

Mice were deeply anesthetized and perfused with 4% paraformaldehyde (PFA) in 0.1 M phosphate buffer (pH 7.4, PB). The fixed brains were immersed in 4% PFA for 2 h and transferred to 30% sucrose in 0.1 M PB. The brains were embedded in the OTC compound (Sakura Finetek Japan). Sections of 30-μm-thickness were prepared by using a cryostat (CM1850, Leica Microsystems) and mounted on MAS-coated glass slides. The sections were incubated in *β*-galactosidase staining solution containing 5 mM potassium hexacyanoferrate (III), 5 mM potassium hexacyanoferrate (II), 2 mM MgCl_2_, and 4% X-gal in PB saline (PBS) at 37°C overnight. The slides were dehydrated in ethanol, and cleared in xylene, and coverslipped with Entellan solution (Merck). The brain sections were viewed by the all-in-one fluorescence microscope (BZ-X800, KEYENCE).

### 2.3 *In situ* hybridization combined with immunohistochemistry

*In situ* hybridization for *Gad67, Drd1, Drd2*, and *c-Fos* mRNAs was performed as described previously (Yamasaki et al., 2010) with minor modification. Plasmids templated for *in vitro* transcription for *Gad67, Drd1 and Drd2* mRNAs were kindly provided by Dr. Masahiko Watanabe, and that for *c-Fos* mRNA was kindly provided by Dr. Haruhiko Bito, and Digoxigenin (DIG)- or fluorescein isothiocyanate (FITC)-labeled cRNA proves were prepared. Fresh-frozen mouse brains were sectioned into 30-μm-thickness slices using a cryostat (CM1850, Leica Microsystems) and mounted on MAS-coated glass slides. Sections were acetylated with 0.25% acetic anhydride in 0.1 M triethanolamine-HCl and hybridization was performed at 63.5°C for 12 h in hybridization buffer supplemented with cRNA probes at a dilution of 1:1000. Post-hybridization washing was at 61°C with standard sodium citrate and formamide. After stringent washing, sections were blocked with DIG blocking solution for 30 min and 0.5% TSA blocking reagent (PerkinElmer) for 30 min. The detection was performed with a peroxidase-conjugated anti-fluorescein antibody (1:1000, 1 h, Invitrogen) followed by incubation with the FITC-TSA plus amplification kit (PerkinElmer) for FITC-labeled cRNA probe or with cyanin 3 (Cy3)-TSA plus amplification kit (PerkinElmer) for DIG-labeled cRNA probe. After inactivation of residual peroxidase activities by dipping sections in 1% H_2_O_2_ for 30 min, sections were subjected to immunostaining.

Immunostaining for phosphorylated S6 (p-S6) was carried out after *in situ* hybridization. Sections were incubated in a blocking solution containing 10% donkey serum and 0.2% Triton X-100 in PBS for 30 min. Sections were immunostained with primary antibody against p-S6 ribosomal protein (Ser235/236) (1:500; #2211, Cell Signaling Technology) overnight at 4°C. The bound antibody was visualized with Cy3 conjugated secondary antibody (1:400; 711-166-152, Jackson ImmunoResearch) or Alexa-488 conjugated secondary antibody (1:400; A11008, Invitrogen) and Hoechst 33258 (SIGMA) was used for staining of cell nuclei. Slides were coverslipped with Vectashield Mounting Medium (H-1000, Vector Laboratories). The brain sections were viewed by the all-in-one fluorescence microscope (BZ-X800, KEYENCE). To analyze horizontal limb of the diagonal band of Broca (HDB) and magnocellular preoptic area (MCPO), we used the right and left sides of a single coronal section at the coordinates of 0.14 mm from bregma. To analyze the other brain regions, we used 2 to 3 coronal sections at the coordinates of 0.5 to 1.18 mm from bregma. Each brain region was identified by aligning the sagittal axis and comparing the sections to the brain atlas (Paxinos and Franklin, 2001). Cell number, fluorescence intensity, and soma size were automatically measured using a hybrid cell count application (BZ-H4C and BZ-H4CM) in the BZ-X analyzer software (BZ-H4A, KEYENCE).

### 2.4 Open field test

The open field test was used to assess anxiety and locomotor activity as described previously with minor modifications (Saito et al., 2020, 2021). A circular arena (diameter: 75 cm) was used with a wall to prevent mice from escaping (height: 35 cm). The area 50 cm from the center of the circle is defined as the inner zone, and the area outside the inner zone is defined as the outer zone. During the test, mice were placed in the center of the circular arena and allowed to explore freely for 10 min under the moderate light condition (30 lux), and the total distance traveled was recorded and scored by Smart V3.0 tracking software (Panlab).

### 2.5 Y-maze test

The Y-maze test was used for the assessment of spatial working memory in mice as described previously (Saito et al., 2020). The Y-maze consists of 3 arms that are the same length and have the same distance from each other (50 cm long, 12 cm high, and 4 cm wide). The light conditions on each arm were adjusted to 35 ± 2 lux. The mouse was placed in the center of the maze and allowed to explore freely for 10 min. The number of arm entries and the number of triads were recorded to calculate the percentage of spontaneous alternation. Spontaneous alternation behavior was defined as the entry into all three arms (i.e., arm A, arm B, and arm C) on consecutive choices in triplet set (i.e., ABC, ACB, BAC, BCA, CAB, and CBA). The percentage of spontaneous alternation behavior was calculated as the percentage of actual alterations to possible alternations, defined as (the total number of arm entries – 2).

### 2.6 Elevated plus maze test

An elevated plus maze test was used to assess anxiety-related behavior in mice as described previously (Saito et al., 2020). The elevated plus maze apparatus consists of two opposing open (25 cm × 8 cm) and two closed arms (25 cm × 8 cm × 20 cm), linked by a neutral area (8 cm × 8 cm) in the center of the apparatus. The entire apparatus was elevated to a height of 5 cm above floor level. The light intensity was adjusted to 20 and 15 lux for open and closed arms, respectively. The mouse was placed in the center of the maze facing the open arm. The mouse was allowed to freely explore the maze for 10 min, and the number of arm entries and the amount of time spent in each arm were recorded.

### 2.7 Rota-rod test

In order to assess motor coordination and motor learning, we performed the rota-rod test, using Rota-Rod Treadmill (MK660C, Muromachi Kikai), as described previously (Sakai et al., 2019). The mouse was placed on a rotating rod with a 30 mm diameter that accelerated from 4 to 40 rpm over 300 s. The maximum observation time was 300 s with constant acceleration, and the latency to fall was recorded. The rod was cleaned by ethanol between the trials. The test was performed on 5 consecutive days under bright-light conditions (approximately 800 lux).

### 2.8 Olfactory habituation and dishabituation test

Olfactory habituation and dishabituation test was performed to assess basic olfactory function and olfactory discrimination (Yang and Crawley, 2009). In this test, acclimation to the test cage was conducted in the separate room from the test room to prevent the mice from exposing the odors before the test. For acclimation to the novel test cage, the mouse was placed in an empty cage and allowed to explore freely for 45 min under the room light conditions (approximately 800 lux). Three odors were presented to the mouse: water (control), vanilla (McCormick), and almond extract (McCormick) (1:100 dilution). Fifty μl of the odors were pipetted onto the tip of the cotton swab. The cotton swab was placed on the wire top of the cage. Each odorant was presented to the mouse 3 times consecutively for 2 min each. Sniffing time was recorded. Active sniffing was defined as the mouse directing its nose 2 cm or closer to the tip of the swab. To analyze the olfactory habituation, the sniffing time of the first and third time of the same odor was compared. To analyze the olfactory dishabituation, the sniffing time was compared between the familiar and novel odors.

### 2.9 Buried food-seeking test

The buried food-seeking test is used to assess olfactory function and odor-induced food-seeking motivation in mice (Machado et al., 2018). Prior to the test, mice were deprived of food for 22 h. Fresh wood bedding was laid in the test cage and 3 g of food pellets were buried 2 cm or 8 cm beneath the surface. The mouse was placed in the cage under room light conditions (approximately 800 lux) and the latency to reach the food was recorded.

### 2.10 Three-chamber odor preference test

Three-chamber odor preference behavior test was conducted as previously described with minor modification (Fitzgerald et al., 2014). Rectangular, three-chambered opaque Plexiglas box (BS-402260, BrainScience Idea) was used. The apparatus is separated into three chambers (each chamber is 20 cm long, 40.5 cm wide, 22 cm high) by transparent plates with the door (50 mm width × 80 mm height). Two plastic cups covered with aluminum foil were placed on either side of the chamber (right and left chamber) to contain the odorants. The light condition was 15 lux. This test consists of 2 sessions: a familiarization session and a test session. During the familiarization session, the mouse was placed in the three-chamber arena and allowed to explore freely for 10 min. During the testing period, 3 g of crushed peanuts were placed in one of two plastic cups. In the other plastic cup, cotton soaked in 300 μL of 10% 2,4,5-trimethylthiazole (TMT) (T1068, TCI Chemical) was placed, and the mouse was allowed to explore freely for 10 min. TMT is a component of fox urine, and it is used to induce unconditioned fear and avoidance in mice (Buron et al., 2007). The amount of time spent in each chamber was recorded.

### 2.11 The *c-Fos* mapping analysis

The *c-Fos* mapping analysis by *in situ* hybridization was performed to detect *c-Fos* expression after the three-chamber odor preference test. The mice that had never performed the behavior tests were divided into two groups, the control group and the experimental group. Three-chamber odor preference tests were performed on the mice in the experimental group as described above. The mice in the control group were placed in the three-chamber with the plastic cups only containing cotton moistened with 300 μL milli-Q water instead of peanuts or 10% TMT. One hour after the experiment, the mice were dissected, and the brain sections were used for the *in situ* hybridization for *c-Fos* mRNA.

### 2.12 Statistical analysis

The significance of differences (*p* < 0.05) was assessed by Welch’s *t*-test for comparison of two groups. In multiple comparison, the significance of differences was evaluated using the analysis of variance (ANOVA) with two-way repeated measure, followed by multiple comparison *post hoc* test; Sidak’s for rota-rod tests and *c-Fos* mapping, and Fisher’s LSD test for olfactory habituation and dishabituation tests and odor preference tests. All data are expressed as the mean ± standard error of the mean (SEM). The detailed statistical methods and values are described in Supplementary Table S1.

## 3 Results

### 3.1 Activation of mTORC1 in striatal MSNs

**Figure 1:**
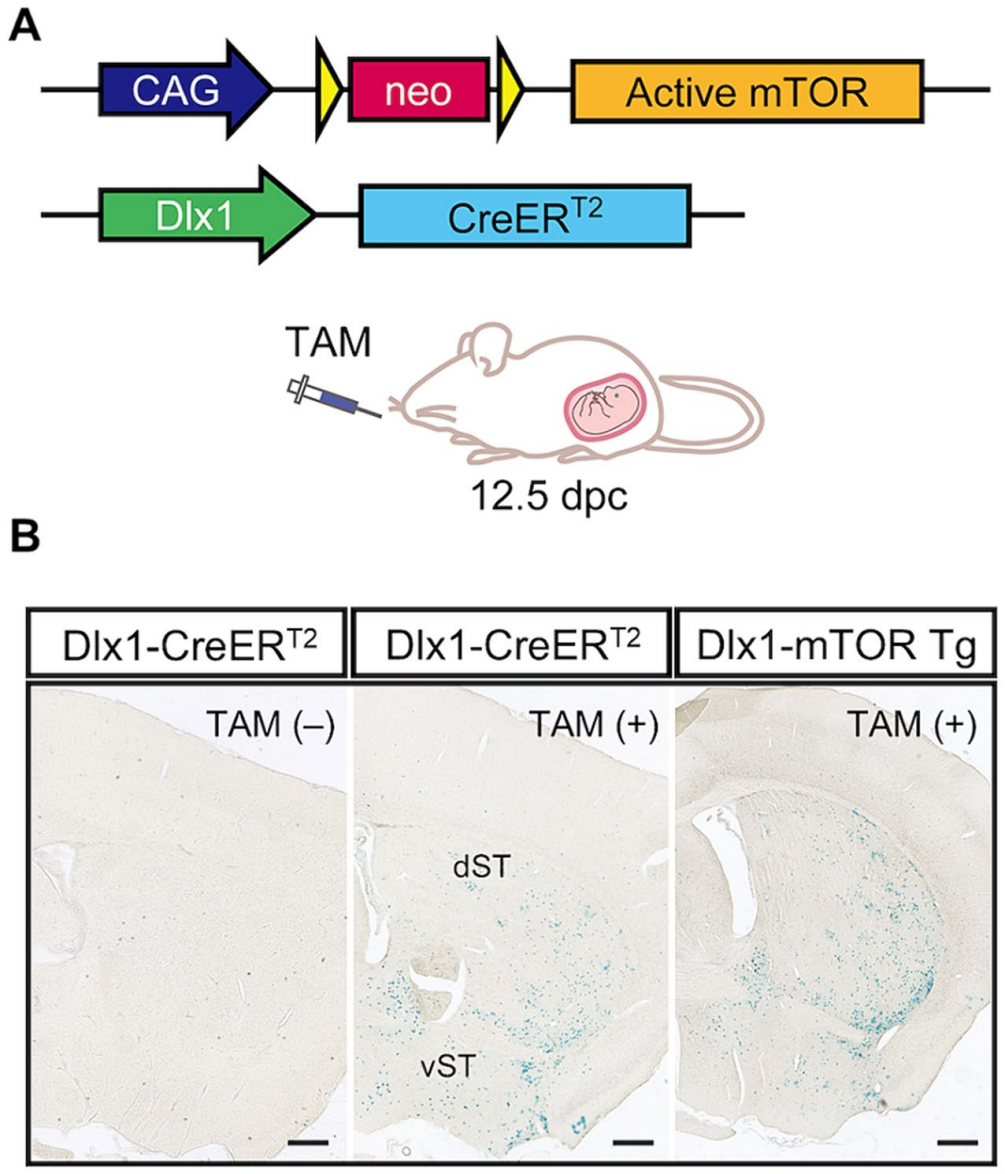
Hyperactivation of the mTORC1 pathway in dorsal and ventral striatum. **(A)** Transgenic strategy for conditional expression of active mTOR kinase by the Cre-*loxP* system. Excision of the floxed *neo* gene by Cre recombinase induces the expression of active mTOR driven by CAG promoter. The *Dlx1* promoter drives CreER^T2^ expression, followed by Cre activation induced by tamoxifen administration at 12.5 dpc. **(B)** Expression pattern of Cre recombinase in Dlx1-CreER^T2^ and Dlx1-mTOR Tg mice. Dlx1-CreER^T2^ and Dlx1-mTOR Tg mice were crossed with Cre-activatable *lacZ* reporter mice (ROSA26R-lacZ), and administered tamoxifen at 12.5 dpc. The brain sections were stained with X-gal. Dlx1-CreER^T2^ mice without tamoxifen administration showed no leaky expression of Cre recombinase. Cre expression was restricted in the dorsal and ventral striatum in both Dlx1-CreER^T2^ and Dlx1-mTOR Tg mice. TAM: tamoxifen. Scale bars, 500 μm.

To examine how activation of mTORC1 pathway affects MSNs in the striatum, we used CAG-mTOR Tg mice in which hyperactive mutant *mTOR^SL1 + IT^* can be conditionally expressed upon excision of the *loxP*-flanked *neo* gene in response to Cre-*loxP* recombination (Kassai et al., 2014; Sakai et al., 2019). The mutant mTOR^SL1 + IT^ can retain its kinase activity toward the mTORC1 pathway but not the mTORC2 pathway under the starvation condition in the cultured cells (Ohne et al., 2008) and brains (Kassai et al., 2014). CAG-mTOR Tg mice were crossed with Dlx1-CreER^T2^ mice to obtain Dlx1-mTOR Tg mice (*Dlx1-CreER^T2^/+; CAG-mTOR^SL1 + IT^/+*) (Figure 1A). Cre recombinase activity was induced by administering tamoxifen to the pregnant mice at 12.5 dpc, when the Dlx1 promoter is highly active in the ventral neural precursors of the ganglionic eminences. The ganglionic eminences contain three subregions; lateral, caudal, and medial ganglionic eminences (LGE, CGE, and MGE). Inhibitory neurons derived from MGE and CGE are distributed in the cerebral cortex, while inhibitory neurons of the striatum and olfactory bulb are derived from LGE. With the single administration of tamoxifen at 12.5 dpc, Cre*-loxP* recombination was expected to induce activation of mTORC1 pathway within the LGE, leaving MGE and CGE unaffected. To visualize tamoxifen-induced Cre*-loxP* recombination in the brain, Dlx1-CreER^T2^ and Dlx1-mTOR Tg mice were crossed with *lacZ* reporter mice (ROSA26R-lacZ mice). Dlx1-CreER^T2^ mice did not show leaky expression of Cre recombinase without tamoxifen administration. Upon tamoxifen administration, both Dlx1-CreER^T2^ and Dlx1-mTOR Tg mice expressed Cre recombinase prominently in the dorsal and ventral striatum leaving the cortical cortex scarcely affected (Figure 1B).

### 3.2 GABAergic neurons are increased in the ventral striatum upon induction of active mTOR

**Figure 2:**
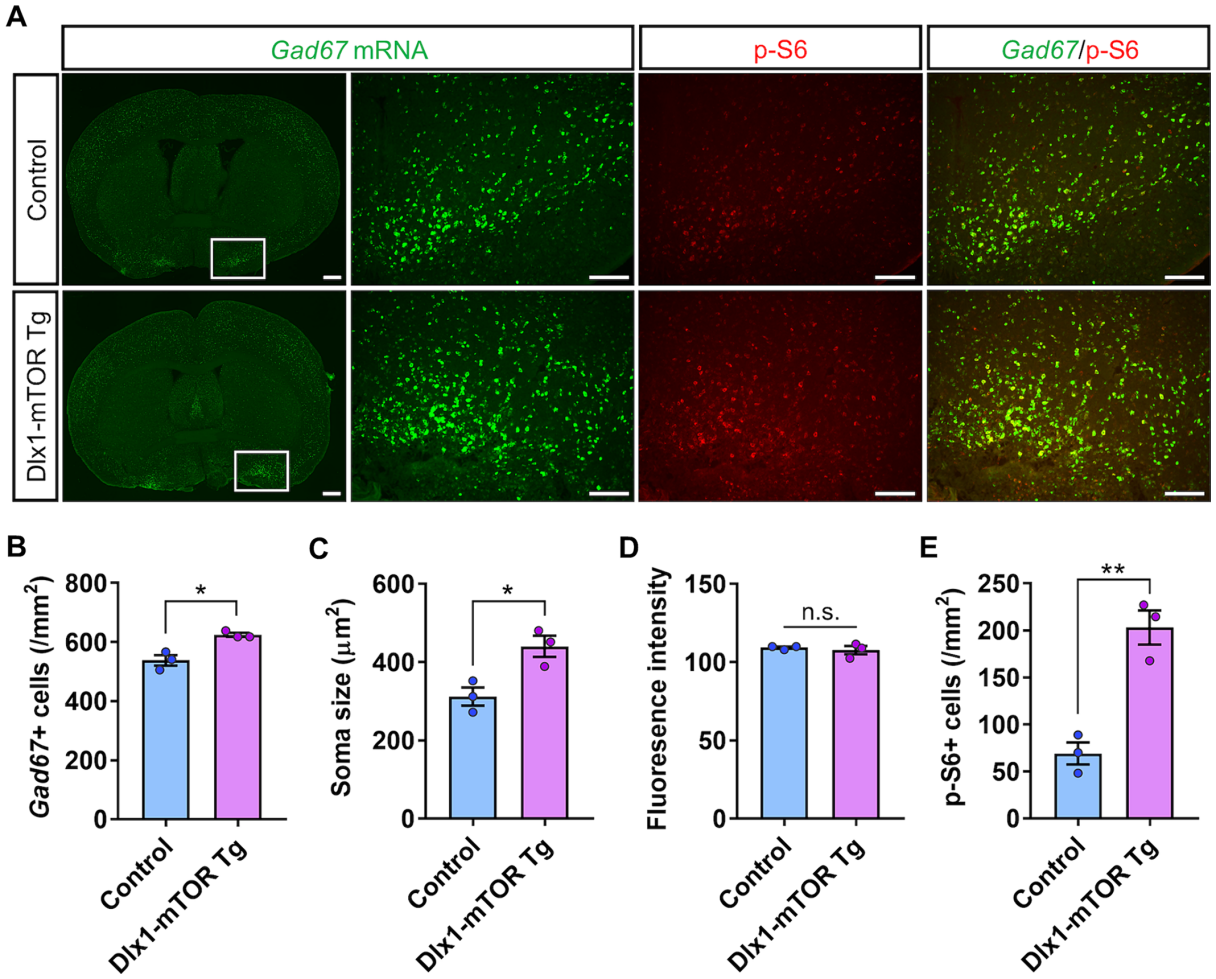
Increase of the cell density of GABAergic neurons by hyperactivation of mTORC1 pathway. **(A)** *In situ* hybridization for *Gad67* (green) and immunohistochemistry of p-S6 (red) in the ventral striatum of control and Dlx1-mTOR Tg mice. The cell density **(B)** and the soma size **(C)** of GABAergic neurons were increased in Dlx1-mTOR Tg mice. **(D)** *Gad67* fluorescence intensity. The cell density of p-S6-positive cells **(E)** was increased in Dlx1-mTOR Tg mice. All data are expressed as mean ± SEM. * *p* < 0.05, **p < 0.01, n.s.: not significant. *p-*value was measured by Welch’s *t*-test. For each analysis, the right and left sides of a brain slice of each mouse were analyzed (*n* = 3). Scale bars, 500 μm (wide angle images); 200 μm (narrow angle images).

**Figure 3:**
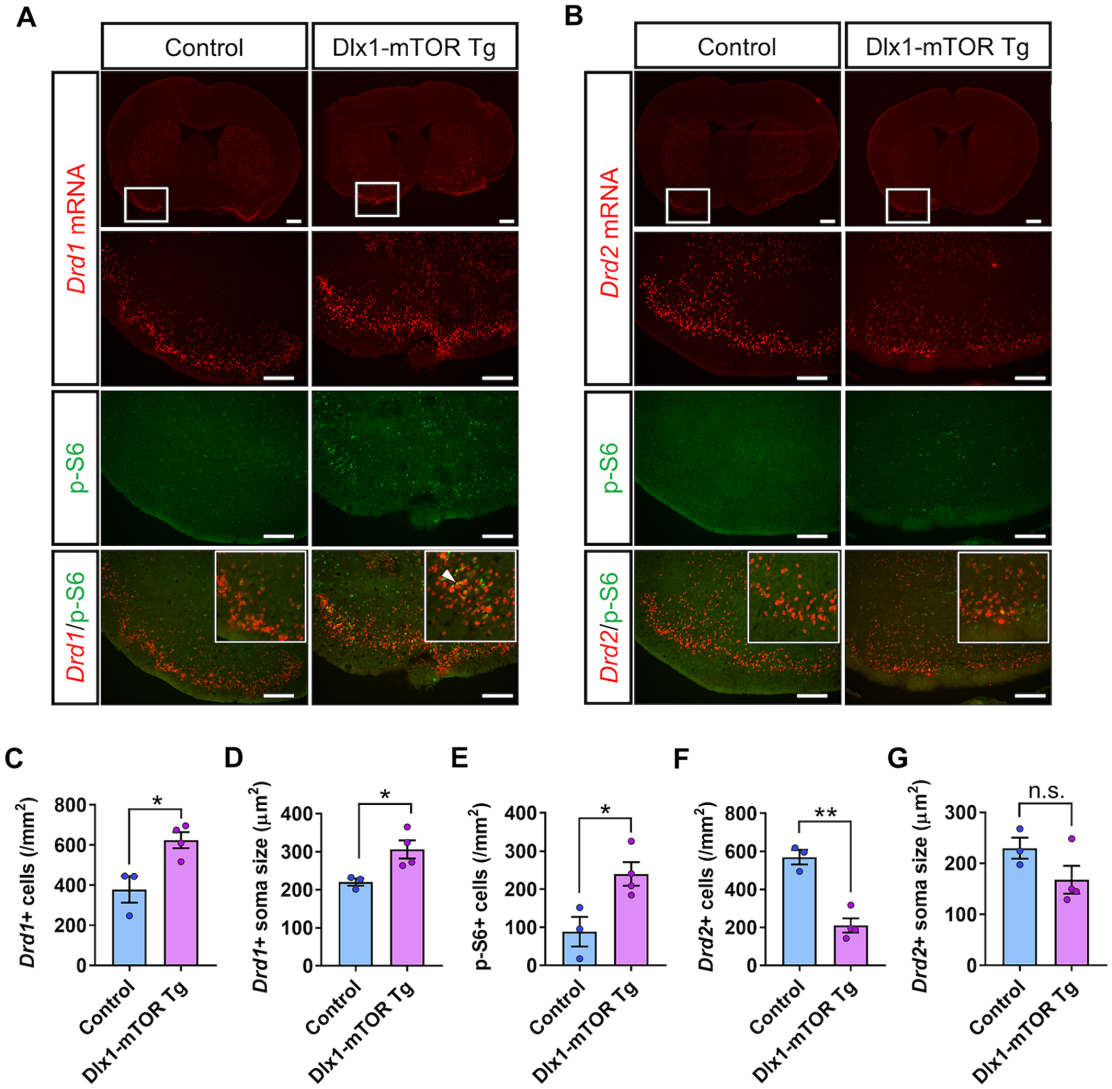
Dysregulation of D1- and D2-expressing MSNs in olfactory tubercle by hyperactivation of mTORC1 pathway. **(A)** *In situ* hybridization of *Drd1* (red) and immunohistochemistry of p-S6 (green) in the olfactory tubercle of control and Dlx1-mTOR Tg mice. A white arrowhead shows *Drd1*/p-S6 double positive cells. **(B)** *In situ* hybridization of *Drd2* (red) and immunohistochemistry of p-S6 (green) in the olfactory tubercle of control and Dlx1-mTOR Tg mice. The cell density **(C)** and the soma size **(D)** of *Drd1*-positive cells were increased in Dlx1-mTOR Tg mice. The cell density of p-S6-positive cells **(E)** was increased in Dlx1-mTOR Tg mice. The cell density of *Drd2*-positive cells **(F)** was decreased in Dlx1-mTOR Tg mice. The soma size of *Drd2*-positive cells **(G)** was not significantly different between both groups. All data are expressed as mean ± SEM. * *p* < 0.05, **p < 0.01, n.s.: not significant. *p-*value was measured by Welch’s *t*-test. For each analysis, 3 brain slices of each mouse were analyzed (*n* = 3 for control, *n* = 4 for Dlx1-mTOR Tg mice). Scale bars, 500 μm (wide angle images); 200 μm (narrow angle images).

Dlx1-mTOR Tg mice are viable with no overt abnormality in their appearances. We first observed the brain morphology and the distribution of Cre-activated cells of Dlx1-mTOR Tg mice using *lacZ* reporter mice. Though active mTOR in excitatory neurons leads to impaired neuronal migration (Kassai et al., 2014), the cell distributions of *lacZ*-positive cells in Dlx1-mTOR Tg mice were not different from those in Dlx1-CreER^T2^ mice (Figure 1B). To perform a detailed analysis on the differences caused by the induction of active mTOR in the striatum, we made histological and behavioral analysis on Dlx1-mTOR Tg mice (*Dlx1-CreER^T2^/+; CAG-mTOR^SL1 + IT^/+*). *CAG-mTOR^SL1 + IT^/+* mice were used as the experimental control mice in the following experiments. We examined the activation of the mTORC1 pathway in the striatum by immunohistochemical analysis for phosphorylation of ribosomal S6 protein, which is used as a readout of mTORC1 activity. The density of p-S6-positive cells was increased in the striatum of Dlx1-mTOR Tg mice (Figures 2E, 3E), while that in the cerebral cortex was not significantly different between Dlx1-mTOR Tg and the control mice (Supplementary Figures S1A,E). To confirm the activation of mTORC1 pathway in the inhibitory neurons, we carried out *in situ* hybridization for *Gad67* mRNA and immunohistochemistry for p-S6 simultaneously in HDB/MCPO, where *Gad67*-positive neurons most densely exist among the ventral striatum (McKenna et al., 2013, Figure 2A). Dlx1-mTOR Tg mice showed increased *Gad67*-positive GABAergic inhibitory neurons compared to the control mice (Figures 2A,B). The soma size of *Gad67*-positive GABAergic inhibitory neurons was also increased in Dlx1-mTOR Tg mice (Figure 2C), while the intensity of *Gad67* mRNA was not significantly changed (Figure 2D). The double staining confirmed that 99% of p-S6-positive mTORC1-hyperactivated neurons were *Gad67*-positive. While *Gad67*-positive GABAergic inhibitory neurons increased in the ventral striatum, the cell density and soma size of *Gad67*-positive GABAergic inhibitory neurons in the cerebral cortex were not significantly different between Dlx1-mTOR Tg and the control mice (Supplementary Figures S1A–C).

### 3.3 Active mTOR dysregulates the expression pattern of the dopamine receptors in MSNs

MSNs expressing *Drd1*and *Drd2* in the striatum are derived from the subventricular zone progenitors of LGE, and Dlx1 serves a regulatory role in the development of the MSNs (Anderson et al., 1997). To investigate the physiological roles of active mTOR in the differentiation of the MSNs, we carried out *in situ* hybridization for *Drd1* and *Drd2* mRNA in the OT (Figure 3). D1- and D2-type MSNs are densely clustered in the OT, and make up 90% of all the OT neurons (Cansler et al., 2020). The number and soma size of *Drd1-*positive MSNs were increased in the OT of Dlx1-mTOR Tg mice (Figures 3A,C,D). p-S6-positive cells were increased in the OT of Dlx1-mTOR Tg mice (Figure 3E) and signals for *Drd1* mRNA and for p-S6 were overlapped (Figure 3A), suggesting that hyperactivation of mTORC1 pathway increases the *Drd1-*positive MSNs. While *Drd1*-positive MSNs were increased, *Drd2*-positive MSNs were decreased in the OT of Dlx1-mTOR Tg mice, compared to the control mice (Figures 3B,F). The *Drd2* signals and p-S6 signals were mutually exclusive (Figure 3B), and the soma size of *Drd2*-positive MSNs was not significantly changed in the OT of Dlx1-mTOR Tg mice (Figure 3G). These results demonstrate that hyperactivation of mTORC1 pathway in MSNs of the OT dysregulates the dopamine receptor expression pattern, leading to the increase of D1-type MSNs and the decrease of D2-type MSNs.

**Figure 4:**
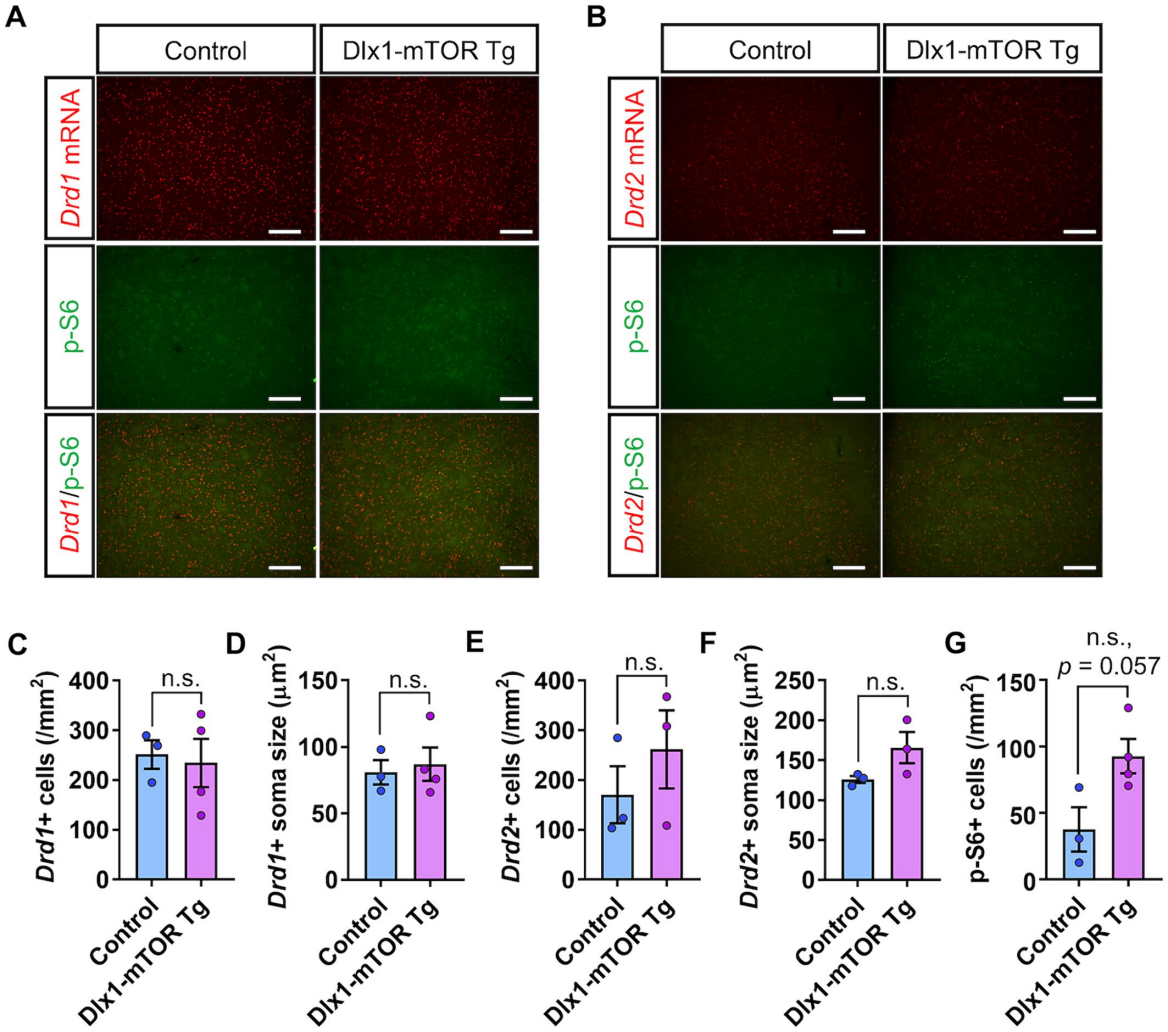
D1- and D2-expressing MSNs in dorsal striatum of Dlx1-mTOR Tg mice. **(A)** *In situ* hybridization of *Drd1* (red) and immunohistochemistry of p-S6 (green) in the dorsal striatum of control and Dlx1-mTOR Tg mice. **(B)** *In situ* hybridization of *Drd2* (red) and immunohistochemistry of p-S6 (green) in the dorsal striatum of control and Dlx1-mTOR Tg mice. The cell density **(C)** and the soma size **(D)** of *Drd1*-positive cells. The cell density **(E)** and the soma size **(F)** of *Drd2*- positive cells. The cell density of p-S6-positive cells **(G)**. All data are expressed as mean ± SEM; n.s.: not significant. *p-*value was measured by Welch’s *t*-test. For each analysis, 3 brain slices of each mouse were analyzed (*n* = 3 for control, *n* = 4 for Dlx1-mTOR Tg mice). Scale bars, 200 μm.

We also analyzed the expression pattern of dopamine receptors in the dorsal striatum of Dlx1-mTOR Tg mice (Figure 4). Though p-S6-positive cells tended to increase in the dorsal striatum of Dlx1-mTOR Tg mice (Figures 4A,B,G, *p* = 0.057), which suggests a potential elevation in mTORC1 activity in this region, no significant differences were observed in cell number or soma size of *Drd1*- and *Drd2*-positive cells between Dlx1-mTOR Tg and the control mice (Figures 4C–F). Taken together, these results demonstrate that hyperactivation of mTORC1 pathway in MSNs dysregulates the dopamine receptor expression pattern in the ventral striatum, while leaving MSNs in the dorsal striatum apparently unaffected. Since we aimed to restrict the Cre-*loxP* recombination to the inhibitory neurons in the striatum, we apply the single administration schedule of tamoxifen at 12.5 dpc. The hyperactivation of mTORC1 pathway in MSNs in dorsal striatum was possibly insufficient to alter the expression of dopamine receptors or make morphological changes, while it might affect the functional differences of MSNs between Dlx1-mTOR Tg and the control mice.

### 3.4 Hyperlocomotion and dysfunction of motor learning in Dlx1-mTOR Tg mice

**Figure 5:**
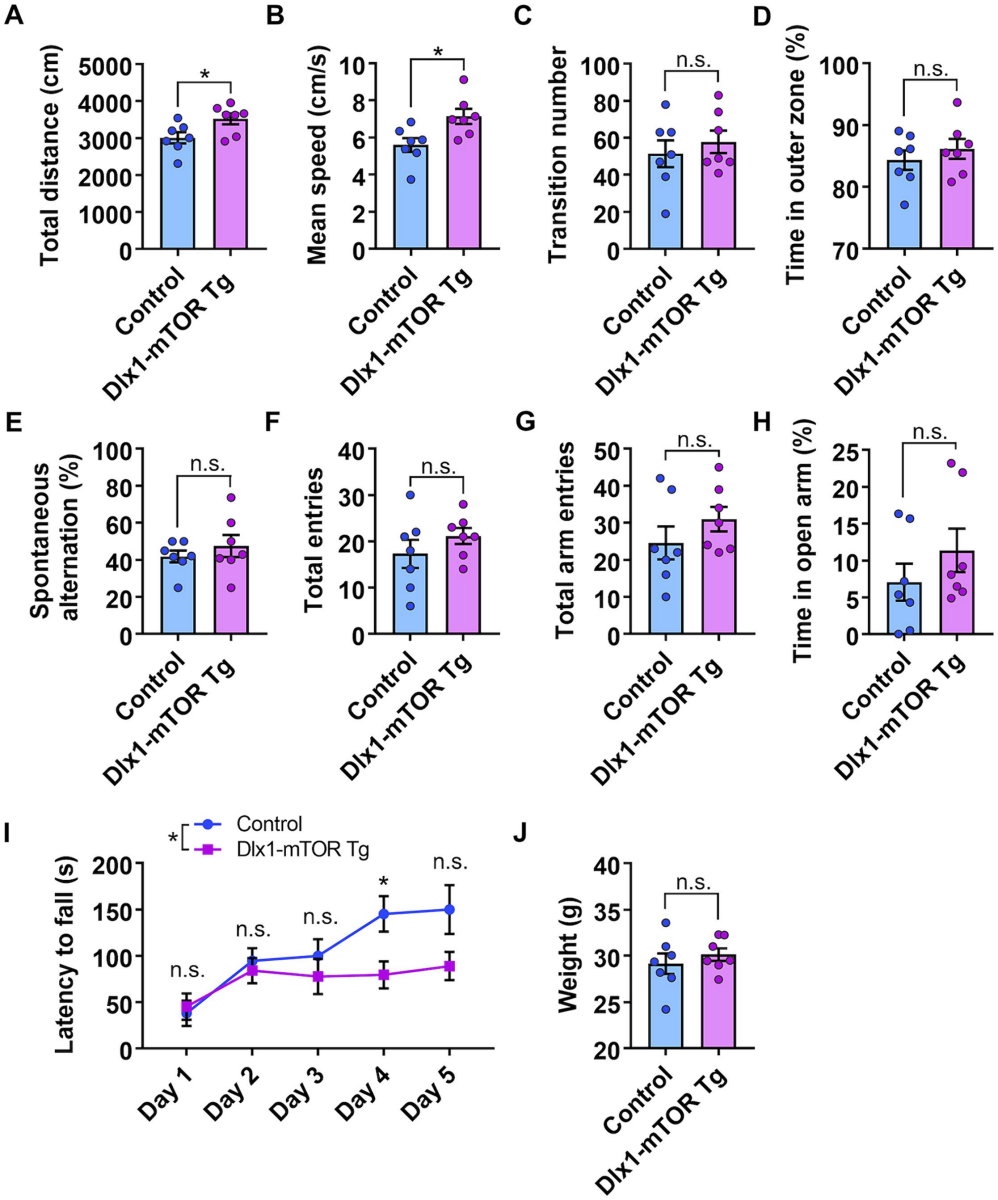
Hyperlocomotion and dysfunction of motor learning in Dlx1-mTOR Tg mice. **(A–D)** Open field test. The total distance traveled **(A)** and the mean speed **(B)** in the open field test were increased in Dlx1-mTOR Tg mice. **(C)** The transition times between outer and inner zones. **(D)** The percentage of time spent in the outer zone. **(E,F)** Y-mase test. The percentage of spontaneous alternation **(E)** and total arm entries **(F)**. **(G,H)** Elevated plus maze test. Total arm entries **(G)** and spent time in the open arm **(H)**. **(I,J)** Rota-rod test. The latency to fall off the rotating rod **(I)** and the body weight at day 1 **(J)**. All data are expressed as mean ± SEM. * *p* < 0.05, n.s.: not significant. *p-*value was measured by Welch’s *t*-test, except for the rota-rod test. For rota-rod test, two-way ANOVA was performed to compare 2 groups, and the *p*-value in each day was analyzed by Sidak’s multiple comparisons test. Each point represents data from an individual male mouse (*n* = 7).

We found that activation of mTORC1 signaling in the MSNs of the striatum resulted in increased cell number and size of GABAergic neurons in the ventral striatum. To investigate how these changes in GABAergic neurons affect mouse behaviors, we performed several behavioral experiments. First, to assess the general locomotor activity and anxiety-like behavior, we conducted the open field test. The total distance traveled and mean speed of locomotion in male Dlx1-mTOR Tg mice were increased compared to the control mice (Figures 5A,B), though only the total distance traveled in Dlx1-mTOR Tg mice was increased in the female cohort (Supplementary Figures S2A,B). These results indicate that the hyperactivation of mTORC1 pathway in the MSNs led to the increased general locomotor activity. The transition number between outer and inter zones was not different between two groups (Figure 5C; Supplementary Figure S2C). The time spent in the outer zone was not changed (Figure 5D; Supplementary Figure S2D), suggesting that Dlx1-mTOR Tg mice did not exhibit anxiety-like behavior. Second, to assess the spatial working memory, we performed Y-maze test. The percentage of spontaneous alternation and the number of total entries were not significantly different in Dlx1-mTOR Tg mice compared to the control mice (Figures 5E,F; Supplementary Figures S2E,F). Next, we conducted an elevated plus maze test to assess anxiety-related behavior in Dlx1-mTOR Tg mice. There were no significant differences in the number of total arm entries or time spent in open arm between Dlx1-mTOR Tg mice and the control mice (Figures 5G,H; Supplementary Figures S2G,H).

Dysregulation of the MSNs in the striatum is associated with the motor dysfunction seen in Parkinson’s and Huntington’s diseases (Chu, 2020; Ehrlich, 2012). Actually, animals with the dysfunction of D1- and D2-type MSNs cause the impairment of the motor behaviors (Bateup et al., 2010; Liang et al., 2022). Therefore, to confirm whether hyperactivation of mTORC1 signaling in the MSNs affects motor coordination and learning, we performed the rota-rod test. Dlx1-mTOR Tg mice showed a significant deficit in the ability to maintain balance on a rotating rod compared to the control mice on day 4, and relatively on day 5 (*p* = 0.07) (Figure 5I), while there was no significant difference in body weight between Dlx1-mTOR Tg and the control mice (Figure 5J). The female cohort also showed significantly shorter time to fall on day 4 and 5 in Dlx1-mTOR Tg mice (Supplementary Figure S2I), though it is difficult to compare Dlx1-mTOR Tg mice to the control mice because of the increased body weight in Dlx1-mTOR Tg mice (Supplementary Figure S2J).

### 3.5 Impaired odor preference behavior in Dlx1-mTOR Tg mice

**Figure 6:**
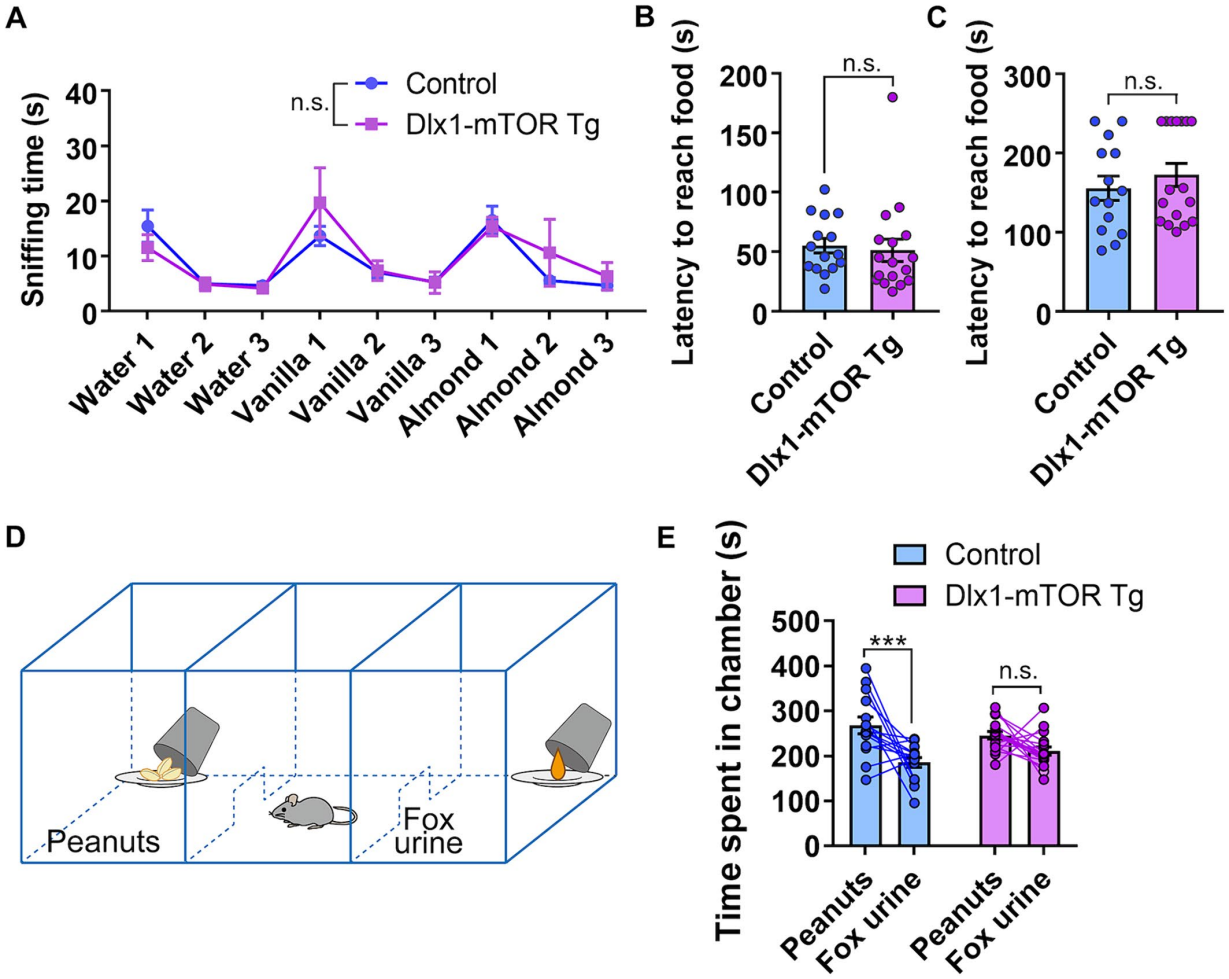
Disruption of odor preference behavior in Dlx1-mTOR Tg mice. **(A)** The sniffing time for each odor in olfactory habituation and dishabituation test. **(B,C)** Buried food-seeking test. The latency to reach the food with 2 cm **(B)** and 8 cm bedding **(C)**. **(D)** Schema of three-chamber odor preference test. **(E)** Time spent in each chamber during three-chamber odor preference test. All data are expressed as mean ± SEM. *** *p* < 0.001, n.s.: not significant. *p-*value was measured by Welch’s *t*-test, except for the olfactory habituation and dishabituation test and the odor preference behavior test. For the olfactory habituation and dishabituation test and the odor preference behavior test, two-way ANOVA followed by Fisher’s LSD multiple comparisons test was used to calculate the *p*-value. Each point represents data from an individual mouse [*n* = 15 for control, *n* = 17 for Dlx1-mTOR Tg mice in **(A–C)**, n = 14 for control, n = 17 for Dlx1-mTOR Tg mice in **(E)**].

Hyperactivation of mTORC1 signaling in the MSNs resulted in increased cell density of inhibitory neurons and p-S6-positive cells in the ventral striatum. The ventral striatum receives direct input from olfactory bulb, and includes many brain areas involved in olfactory behaviors, such as nucleus accumbens, OT and HDB/MCPO. To assess whether the active mTOR in MSNs affects the olfactory function, we carried out several olfactory behavior tests. First, we performed the olfactory habituation and dishabituation test to measure the basic olfaction and olfactory discrimination behavior in Dlx1-mTOR Tg mice. When we repeatedly presented the same odor to Dlx1-mTOR Tg mice, the sniffing time was gradually decreased within each trial (Figure 6A, *p* < 0.05). On the other hand, when a novel odor was presented, the sniffing time was increased compared to familiar odors (Figure 6A, *p* < 0.05). These results indicated that the basic olfactory function and olfactory discrimination behavior were not impaired in Dlx-mTORC1 Tg mice.

The OT interconnects with amygdala, thalamus, hypothalamus, hippocampus, and brain stem, and is considered as an important area for multi-sensory processing (Wesson and Wilson, 2011). For instance, the OT is involved in the food-seeking-related motivation, odor preference and reward cognition (Murata et al., 2015; Fitzgerald et al., 2014). Therefore, we carried out the buried food-seeking test to assess the odor-induced food-seeking motivation of Dlx1-mTOR Tg mice. There was no significant difference in the latency to reach the reward food between Dlx1-mTORC1 Tg and the control mice (Figures 6B,C). Our results suggest that the odor-induced food-seeking motivation of Dlx1-mTOR Tg mice was not affected by active mTOR. Next, to examine the influence of mTOR activation in MSNs on odor preference behavior, we performed three-chamber odor preference test (Figure 6D). In general, mice prefer peanuts odor and avoid the TMT odor, a component of fox urine, and therefore, mice tend to spend more time in the chamber with peanuts. In the control mice, time spent in the peanut chamber was significantly longer than that in the TMT chamber (Figure 6E). However, in Dlx1-mTOR Tg mice, there was no significant difference between the time spent in the peanut and TMT chambers. These results suggest that the preference for the peanut chamber was abolished in Dlx1-mTOR Tg mice.

### 3.6 Olfactory circuit in piriform cortex was altered in Dlx1-mTOR Tg mice

**Figure 7:**
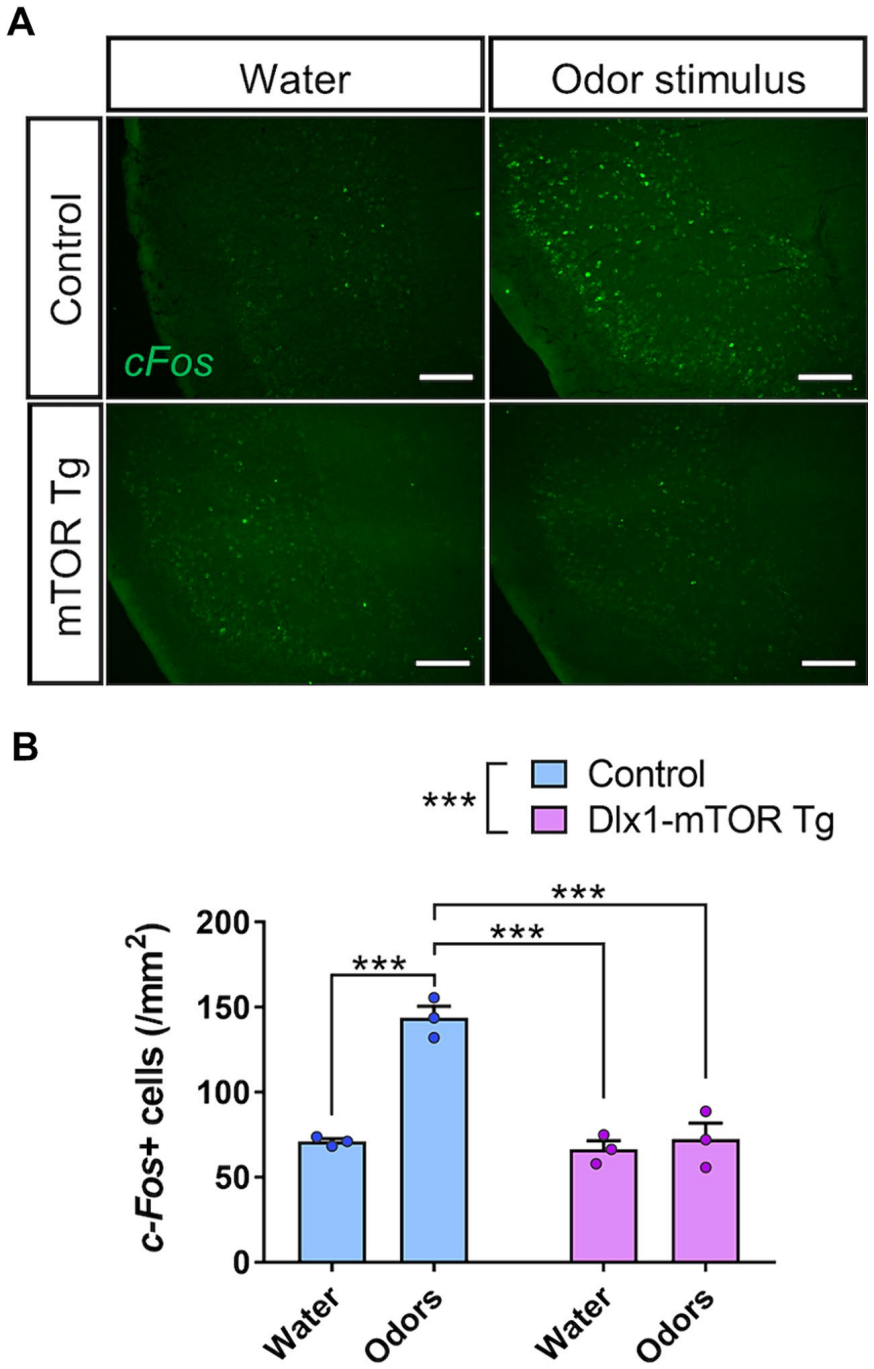
Impairment of olfactory circuit in piriform cortex of Dlx1-mTOR Tg mice. **(A)** *In situ* hybridization of *c-Fos* in the piriform cortex of the control and Dlx1-mTOR Tg mice. Neuronal activity was increased by odors in control but not in Dlx1-mTOR Tg mice. **(B)** The cell density of *c-Fos*-positive cells in the piriform cortex. All data are expressed as mean ± SEM. *** *p* < 0.001, n.s.: not significant. Two-way ANOVA followed by Sidak’s multiple comparisons test was used to calculate the *p*-value. For the analysis, 2 brain slices of each mouse were analyzed (*n* = 3). Scale bars, 200 μm.

To assess the alteration of neural circuit in odor preference behavior, *in situ* hybridization of *c-Fos* in the olfactory cortex was performed after three-chamber odor preference test. Dlx1-mTOR Tg and the control mice were divided into two groups, the odor-guided experimental group and the odor-free control group. The three-chamber odor preference tests were performed using peanut and 10% TMT for the odor-guided group and using only water for the odor-free group. The control mice in odor-guided experimental group showed increased *c-Fos* signals in piriform cortex but not in other olfactory cortex areas including the OT. No significant difference was detected between the odor-guided and odor-free groups of Dlx1-mTOR Tg mice (Figures 7A,B). These results indicate that hyperactivation of mTORC1 pathway in MSNs altered the neuronal activation in the piriform cortex during the odor preference behavior.

## 4 Discussion

In this study, we demonstrated that hyperactivation of mTORC1 signaling has a significant impact on cell size regulation and dopamine receptor expression patterns in striatal MSNs. We also showed the impaired motor learning and disrupted odor preference behavior in Dlx1-mTOR Tg mice. Previous animal models for mTOR activation in inhibitory neurons have shown enlarged soma size and disrupted functional development of the cortical interneurons (Fu et al., 2012; Malik et al., 2019; Amegandjin et al., 2021). Morphological and functional impairment of inhibitory neurons of the striatum of Dlx1-mTOR Tg mice were consistent with these results. Mice with SST-specific and PV-specific conditional deletion of *Pten* gene demonstrated impaired motor learning and social deficits (Shin et al., 2021). Impaired motor learning in Dlx1-mTOR Tg mice was consistent with this result. However, our animal model of striatal specific hyperactivation of mTOR showed several different phenotypes including olfactory preference behavior from the other models.

With our tamoxifen administration schedule, Dlx1-mTOR Tg mice showed maximum induction of Cre-*loxP* recombination and resulted in highly abnormal MSNs in the ventral striatum. Ventral striatum not only functions as the center of reward-guided behaviors but also has the pivotal role in olfactory-related behaviors. We found that sustained activation of mTORC1 pathway impaired odor preference behavior, leaving the fundamental sensing of olfaction preserved. As D1- and D2-type MSNs in OT regulate odor-attractive and odor-aversive behaviors, respectively (Murata, 2020), dysregulated expression of dopamine receptors seems to have caused loss of preference for peanuts odor against TMT. Moreover, *in situ* hybridization analysis for *c-Fos* showed that the neuronal activation of the piriform cortex during the odor preference behavior was inhibited in Dlx1-mTOR Tg mice. Though the neural circuits underlying the olfactory preference behavior are still unrevealed, the results provide fascinating insights into the possible projection of the striatal MSNs to the piriform cortex regulating the odor preference behavior. The piriform cortex is the largest component of the olfactory cortex and OT has the massive input proportion from piriform cortex (Bekkers and Suzuki, 2013). Though the reciprocal connections between piriform cortex and the other olfactory cortex, including OT, have been revealed (Zhang et al., 2017; Wang et al., 2020), the function of the neuronal circuits between piriform cortex and the other olfactory cortex are still unclear. Further investigation is needed to elucidate the neuronal mechanism underlying the olfactory preference behavior.

Hyperactivation of mTOR in Dlx1-mTOR Tg mice increases D1-type MSNs and decreases D2-type MSNs in the ventral striatum. Further, MSNs in the dorsal striatum might have been affected in Dlx1-mTOR Tg mice, since p-S6 positive cells were relatively increased in the dorsal striatum. Dysregulation of D1- and D2-type MSNs in dorsal striatum is reported in patients harboring neurodegenerative diseases including Parkinson’s disease and Huntington’s disease (Stephens et al., 2005; Cicchetti et al., 2000). To examine the function of D1- and D2-type MSNs, several animal models were generated. Conditional KO of dopamine- and cAMP-regulated phosphoprotein Mr. 32 kDa (DARPP-32), which regulates dopamine signaling, in D1- and D2-type MSNs both altered motor behaviors (Bateup et al., 2010). While D1-expressing cell-specific DARPP-32 KO mice showed decreased basal locomotor activity, D2-expressing cell-specific DARPP-32 KO mice showed increased basal locomotor activity, which was consistent with our results showing the increased total distance traveled and mean speed of locomotion in Dlx1-mTOR Tg mice in open field test. D1- and D2-type MSNs are also important in motor learning, which was indicated by the impaired locomotor activity and motor learning in whole-body D1 and D2 KO mice (Nakamura et al., 2014). Further, conditional KO of D2 in indirect pathway projecting MSNs showed dysfunction of motor and other learning skills (Augustin et al., 2020), which was consistent with our results showing the impaired performance of Dlx1-mTOR Tg mice on the rota-rod test. Though the further investigation is indispensable for understanding the mechanism, impaired motor learning of Dlx1-mTOR Tg mice might have been caused by the impaired function of MSNs in dorsal striatam.

The striatum-specific hyperactivation of mTOR revealed that mTORC1 signaling plays an important role in morphological and functional development of MSNs and the dysregulated MSNs caused the impaired motor learning and odor preference. Though the dysregulated MSNs in dorsal striatum and the motor abnormalities have been already reported in patients with Parkinson’s disease and Huntington’s disease, our study showed the pivotal insight into the contribution of mTORC1 pathway to the motor abnormalities in neurodegenerative diseases. Further, our animal model showed impaired odor preference. The hyperactivation of mTORC1 pathway occurs in the inhibitory neurons of TSC patients exhibiting neuropsychiatric symptoms such as ASD. Our results imply the relationships between the sensory defects in mTOR-related neuropsychiatric disease and activation of mTORC1 pathway in the inhibitory neurons of the striatum. Further investigation is needed to elucidate the underlying mechanism.

## Data Availability

The raw data supporting the conclusions of this article will be made available by the authors, without undue reservation.

## References

[ref1] Albert V. Hall M. N. (2015). mTOR signaling in cellular and organismal energetics. Curr. Opin. Cell Biol. 33, 55–66. doi: 10.1016/j.ceb.2014.12.001, PMID: 25554914

[ref2] Amegandjin C. A. Choudhury M. Jadhav V. Carriço J. N. Quintal A. Berryer M.. (2021). Sensitive period for rescuing parvalbumin interneurons connectivity and social behavior deficits caused by TSC1 loss. Nat. Commun. 12:3653. doi: 10.1038/s41467-021-23939-7, PMID: 34135323 PMC8209106

[ref3] Anderson S. A. Qiu M. Bulfone A. Eisenstat D. D. Meneses J. Pederson R.. (1997). Mutations of the homeobox genes Dlx-1 and Dlx-2 disrupt the striatal subventricular zone and differentiation of late born striatal neurons. Neuron 19, 27–37. doi: 10.1016/s0896-6273(00)80345-1, PMID: 9247261

[ref4] Augustin S. M. Loewinger G. C. O’Neal T. J. Kravitz A. V. Lovinger D. M. (2020). Dopamine D2 receptor signaling on iMSNs is required for initiation and vigor of learned actions. Neuropsychopharmacology 45, 2087–2097. doi: 10.1038/s41386-020-00799-1, PMID: 32811899 PMC7547091

[ref5] Bateup H. S. Santini E. Shen W. Birnbaum S. Valjent E. Surmeier D. J.. (2010). Distinct subclasses of medium spiny neurons differentially regulate striatal motor behaviors. Proc. Natl. Acad. Sci. USA 107, 14845–14850. doi: 10.1073/pnas.1009874107, PMID: 20682746 PMC2930415

[ref6] Benthall K. N. Cording K. R. Agopyan-Miu A. H. C. W. Wong C. D. Chen E. Y. Bateup H. S. (2021). Loss of Tsc1 from striatal direct pathway neurons impairs endocannabinoid-LTD and enhances motor routine learning. Cell Rep. 36:109511. doi: 10.1016/j.celrep.2021.109511, PMID: 34380034 PMC8404511

[ref7] Bekkers J. M. Suzuki N. (2013). Neurons and circuits for odor processing in the piriform cortex. Trends Neurosci. 36, 429–438. doi: 10.1016/j.tins.2013.04.005, PMID: 23648377

[ref8] Bové J. Martínez-Vicente M. Vila M. (2011). Fighting neurodegeneration with rapamycin: mechanistic insights. Nat. Rev. Neurosci. 12, 437–452. doi: 10.1038/nrn3068, PMID: 21772323

[ref9] Buron G. Hacquemand R. Pourie G. Lucarz A. Jacquot L. Brand G. (2007). Comparative behavioral effects between synthetic 2,4,5-trimethylthiazoline (TMT) and the odor of natural fox (*Vulpes vulpes*) feces in mice. Behav. Neurosci. 121, 1063–1072. doi: 10.1037/0735-7044.121.5.1063, PMID: 17907837

[ref10] Busquets-Garcia A. Gomis-González M. Guegan T. Agustín-Pavón C. Pastor A. Mato S.. (2013). Targeting the endocannabinoid system in the treatment of fragile X syndrome. Nat. Med. 19, 603–607. doi: 10.1038/nm.3127, PMID: 23542787

[ref11] Cansler H. L. Wright K. N. Stetzik L. A. Wesson D. W. (2020). Neurochemical organization of the ventral striatum's olfactory tubercle. J. Neurochem. 152, 425–448. doi: 10.1111/jnc.14919, PMID: 31755104 PMC7042089

[ref12] Chu H.-Y. (2020). Synaptic and cellular plasticity in Parkinson's disease. Acta Pharmacol. Sin. 41, 447–452. doi: 10.1038/s41401-020-0371-0, PMID: 32112041 PMC7470833

[ref13] Cicchetti F. Prensa L. Wu Y. Parent A. (2000). Chemical anatomy of striatal interneurons in normal individuals and in patients with Huntington’s disease. Brain Res. Brain Res. Rev. 34, 80–101. doi: 10.1016/s0165-0173(00)00039-4, PMID: 11086188

[ref14] Ehrlich M. E. (2012). Huntington's disease and the striatal medium spiny neuron: cell-autonomous and non-cell-autonomous mechanisms of disease. Neurotherapeutics 9, 270–284. doi: 10.1007/s13311-012-0112-2, PMID: 22441874 PMC3337013

[ref15] Fitzgerald B. J. Richardson K. Wesson D. W. (2014). Olfactory tubercle stimulation alters odor preference behavior and recruits forebrain reward and motivational centers. Front. Behav. Neurosci. 8:81. doi: 10.3389/fnbeh.2014.00081, PMID: 24672445 PMC3954079

[ref16] Foster K. G. Fingar D. C. (2010). Mammalian target of rapamycin (mTOR): conducting the cellular signaling symphony. J. Biol. Chem. 285, 14071–14077. doi: 10.1074/jbc.R109.094003, PMID: 20231296 PMC2863215

[ref17] Fu C. Cawthon B. Clinkscales W. Bruce A. Winzenburger P. Ess K. C. (2012). GABAergic interneuron development and function is modulated by the Tsc1 gene. Cereb. Cortex 22, 2111–2119. doi: 10.1093/cercor/bhr300 22021912 PMC3412444

[ref18] Ikemoto S. (2007). Dopamine reward circuitry: two projection systems from the ventral midbrain to the nucleus accumbens-olfactory tubercle complex. Brain Res. Rev. 56, 27–78. doi: 10.1016/j.brainresrev.2007.05.004, PMID: 17574681 PMC2134972

[ref19] Jaworski J. Sheng M. (2006). The growing role of mTOR in neuronal development and plasticity. Mol. Neurobiol. 34, 205–219. doi: 10.1385/MN:34:3:205, PMID: 17308353

[ref20] Kassai H. Sugaya Y. Noda S. Nakao K. Maeda T. Kano M.. (2014). Selective activation of mTORC1 signaling recapitulates microcephaly, tuberous sclerosis, and neurodegenerative diseases. Cell Rep. 7, 1626–1639. doi: 10.1016/j.celrep.2014.04.048, PMID: 24857653

[ref21] Laplante M. Sabatini D. M. (2012). mTOR signaling in growth control and disease. Cell 149, 274–293. doi: 10.1016/j.cell.2012.03.017, PMID: 22500797 PMC3331679

[ref22] Liang B. Zhang L. Zhang Y. Werner C. T. Beacher N. J. Denman A. J.. (2022). Striatal direct pathway neurons play leading roles in accelerating rotarod motor skill learning. iScience. 25:104245. doi: 10.1016/j.isci.2022.104245, PMID: 35494244 PMC9046249

[ref23] Machado C. F. Reis-Silva T. M. Lyra C. S. Felicio L. F. Malnic B. (2018). Buried food-seeking test for the assessment of olfactory detection in mice. Bio. Protoc. 8:e2897. doi: 10.21769/BioProtoc.2897, PMID: 34286006 PMC8275211

[ref24] Malik R. Pai E. L.-L. Rubin A. N. Stafford A. M. Angara K. Minasi P.. (2019). Tsc1 represses parvalbumin expression and fast-spiking properties in somatostatin lineage cortical interneurons. Nat. Commun. 10:4994. doi: 10.1038/s41467-019-12962-4, PMID: 31676823 PMC6825152

[ref25] McKenna J. T. Yang C. Franciosi S. Winston S. Abarr K. K. Rigby M. S.. (2013). Distribution and intrinsic membrane properties of basal forebrain GABAergic and parvalbumin neurons in the mouse. J. Comp. Neurol. 521, 1225–1250. doi: 10.1002/cne.23290, PMID: 23254904 PMC3627393

[ref26] Murata K. Kanno M. Ieki N. Mori K. Yamaguchi M. (2015). Mapping of learned odor-induced motivated behaviors in the mouse olfactory tubercle. J. Neurosci. 35, 10581–10599. doi: 10.1523/JNEUROSCI.0073-15.2015, PMID: 26203152 PMC6605114

[ref27] Murata K. (2020). Hypothetical roles of the olfactory tubercle in odor-guided eating behavior. Front. Neural. Circuits. 14:577880. doi: 10.3389/fncir.2020.577880, PMID: 33262693 PMC7686465

[ref28] Nakamura T. Sato A. Kitsukawa T. Momiyama T. Yamamori T. Sasaoka T. (2014). Distinct motor impairments of dopamine D1 and D2 receptor knockout mice revealed by three types of motor behavior. Front. Integr. Neurosci. 8:56. doi: 10.3389/fnint.2014.00056, PMID: 25076876 PMC4097398

[ref29] Ohne Y. Takahara T. Hatakeyama R. Matsuzaki T. Noda M. Mizushima N.. (2008). Isolation of hyperactive mutants of mammalian target of rapamycin. J. Biol. Chem. 283, 31861–31870. doi: 10.1074/jbc.M801546200, PMID: 18812319

[ref30] Orlova K. A. Crino P. B. (2010). The tuberous sclerosis complex. Ann. N. Y. Acad. Sci. 1184, 87–105. doi: 10.1111/j.1749-6632.2009.05117.x, PMID: 20146692 PMC2892799

[ref31] Paxinos G. Franklin K. B. J. (2001). The mouse brain in stereotaxic coordinates. 2nd Edn. Cambridge, MA: Academic Press.

[ref32] Pryor W. M. Biagioli M. Shahani N. Swarnkar S. Huang W.-C. Page D. T.. (2014). Huntingtin promotes mTORC1 signaling in the pathogenesis of Huntington's disease. Sci. Signal. 7:ra103. doi: 10.1126/scisignal.2005633, PMID: 25351248

[ref33] Saito R. Koebis M. Nagai T. Shimizu K. Liao J. Wulaer B.. (2020). Comprehensive analysis of a novel mouse model of the 22q11.2 deletion syndrome: a model with the most common 3.0-Mb deletion at the human 22q11.2 locus. Transl. Psychiatry 10:35. doi: 10.1038/s41398-020-0723-z, PMID: 32066675 PMC7026107

[ref34] Saito R. Miyoshi C. Koebis M. Kushima I. Nakao K. Mori D.. (2021). Two novel mouse models mimicking minor deletions in 22q11.2 deletion syndrome revealed the contribution of each deleted region to psychiatric disorders. Mol. Brain 14:68. doi: 10.1186/s13041-021-00778-7, PMID: 33845872 PMC8042712

[ref35] Sakai Y. Kassai H. Nakayama H. Fukaya M. Maeda T. Nakao K.. (2019). Hyperactivation of mTORC1 disrupts cellular homeostasis in cerebellar Purkinje cells. Sci. Rep. 9:2799. doi: 10.1038/s41598-019-38730-4, PMID: 30808980 PMC6391425

[ref36] Santini E. Heiman M. Greengard P. Valjent E. Fisone G. (2009). Inhibition of mTOR signaling in Parkinson's disease prevents L-DOPA-induced dyskinesia. Sci. Signal. 2:ra36. doi: 10.1126/scisignal.2000308, PMID: 19622833

[ref37] Sato A. Kasai S. Kobayashi T. Takamatsu Y. Hino O. Ikeda K.. (2012). Rapamycin reverses impaired social interaction in mouse models of tuberous sclerosis complex. Nat. Commun. 3:1292. doi: 10.1038/ncomms2295, PMID: 23250422 PMC3535343

[ref38] Shin S. Santi A. Huang S. (2021). Conditional Pten knockout in parvalbumin- or somatostatin-positive neurons sufficiently leads to autism-related behavioral phenotypes. Mol. Brain 14:24. doi: 10.1186/s13041-021-00731-8, PMID: 33504340 PMC7839207

[ref39] Soriano P. (1999). Generalized lacZ expression with the ROSA26 Cre reporter strain. Nat. Genet. 21, 70–71. doi: 10.1038/5007 9916792

[ref40] Stephens B. Mueller A. J. Shering A. F. Hood S. H. Taggart P. Arbuthnott G. W.. (2005). Evidence of a breakdown of corticostriatal connections in Parkinson’s disease. Neuroscience 132, 741–754. doi: 10.1016/j.neuroscience.2005.01.007 15837135

[ref41] Taniguchi H. He M. Wu P. Kim S. Paik R. Sugino K.. (2011). A resource of Cre driver lines for genetic targeting of GABAergic neurons in cerebral cortex. Neuron 71, 995–1013. doi: 10.1016/j.neuron.2011.07.026, PMID: 21943598 PMC3779648

[ref42] Wang L. Zhang Z. Chen J. Manyande A. Haddad R. Liu Q.. (2020). Cell-type-specific whole-brain direct inputs to the anterior and posterior piriform cortex. Front. Neural. Circuits. 14:4. doi: 10.3389/fncir.2020.00004, PMID: 32116571 PMC7019026

[ref43] Wesson D. W. Wilson D. A. (2011). Sniffing out the contributions of the olfactory tubercle to the sense of smell: hedonics, sensory integration, and more? Neurosci. Biobehav. Rev. 35, 655–668. doi: 10.1016/j.neubiorev.2010.08.004, PMID: 20800615 PMC3005978

[ref44] Yager L. M. Garcia A. F. Wunsch A. M. Ferguson S. M. (2015). The ins and outs of the striatum: role in drug addiction. Neuroscience 301, 529–541. doi: 10.1016/j.neuroscience.2015.06.033, PMID: 26116518 PMC4523218

[ref45] Yamasaki M. Matsui M. Watanabe M. (2010). Preferential localization of muscarinic M1 receptor on dendritic shaft and spine of cortical pyramidal cells and its anatomical evidence for volume transmission. J. Neurosci. 30, 4408–4418. doi: 10.1523/JNEUROSCI.5719-09.2010, PMID: 20335477 PMC6634497

[ref46] Yang M. Crawley J. N. (2009). Simple behavioral assessment of mouse olfaction. Curr. Protoc. Neurosci. 8, Unit 8.24–Unit 8.12. doi: 10.1002/0471142301.ns0824s48, PMID: 19575474 PMC2753229

[ref47] Zhang Z. Zhang H. Wen P. Zhu X. Wang L. Liu Q.. (2017). Whole-brain mapping of the inputs and outputs of the medial part of the olfactory tubercle. Front. Neural. Circuits 11:52. doi: 10.3389/fncir.2017.00052, PMID: 28804450 PMC5532451

