# Supplementary material for: Hyperactive mTORC1 in striatum dysregulates dopamine receptor expression and odor preference behavior
Source: Front Neurosci. 2024 Aug 30;18:1461178. doi: 10.3389/fnins.2024.1461178 (PMC11392874; doi:10.3389/fnins.2024.1461178)
Supplement: Supplementary file 1 [file Presentation_1.pdf]

Supplementary Material

1 Supplementary Figures

Supplementary Figure S1

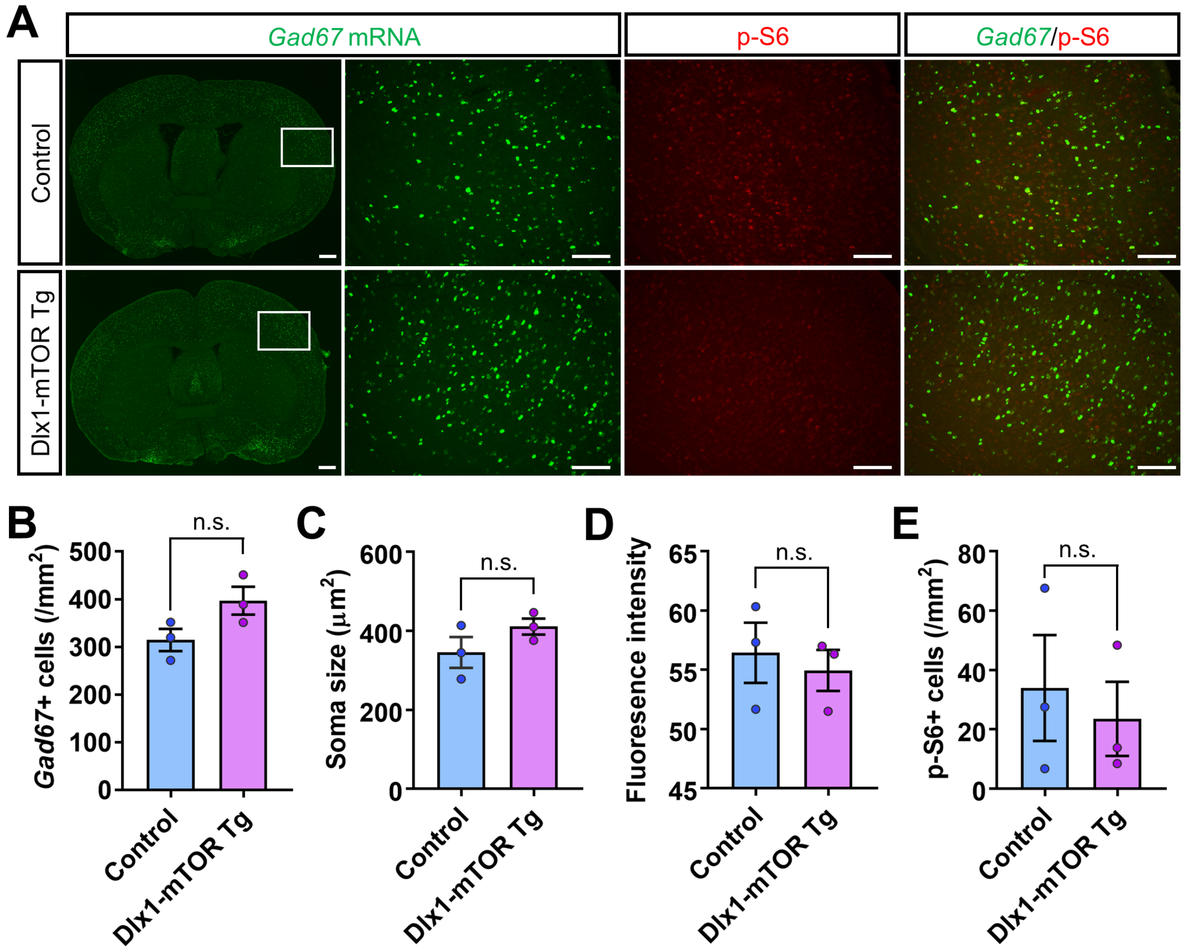

Supplementary Figure S1. GABAergic neurons in cerebral cortex in Dlx1-mTOR Tg mice.

(A) *In situ* hybridization for *Gad67* (green) and immunohistochemistry of p-S6 (red) in the cerebral cortex of control and Dlx1-mTOR Tg mice. The cell density (B) and the soma size (C) of GABAergic neurons and *Gad67* fluorescence intensity (D) in Dlx1-mTOR Tg mice were not significantly different from those in control mice. The cell density of p-S6-positive cells (E) was not significantly different between control and Dlx1-mTOR Tg mice. All data are expressed as mean ± SEM. n.s.: not significant. *P*-value was measured by Welch's *t*-test. For each analysis, 3 brain slices of each mouse were analyzed (n = 3). Scale bars, 500 μm (wide angle images); 200 μm (narrow angle images).

Supplementary Figure S2

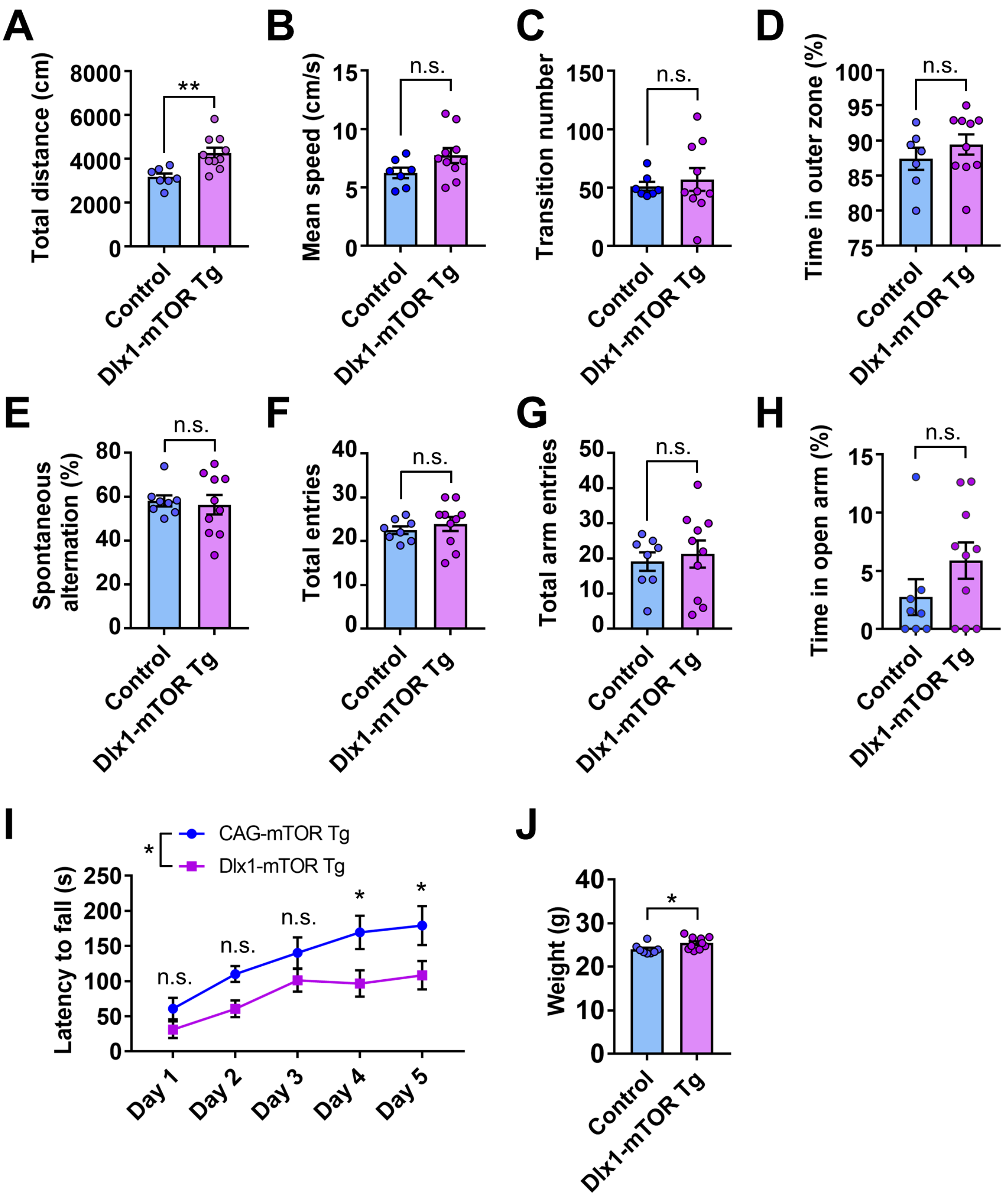

Supplementary Figure S2. Behavioral tests in female Dlx1-mTOR Tg mice.

(A-D) Open field test. The total distance traveled (A) in the open field test was increased in female Dlx1-mTOR Tg mice. (B) The mean speed. (C) The transition times between outer and inner zones. (D) The percentage of time spent in the outer zone. (E, F) Y-mase test. The percentage of spontaneous alternation (E) and total arm entries (F). (G, H) Elevated plus maze test. Total arm entries (G) and spent time in the open arm (H). (I, J) Rota-rod test. The latency to fall off the rotating rod (I) and the body weight at day 1 (J). All data are expressed as mean ± SEM. * $p < 0.05$, ** $p < 0.01$, n.s.: not significant. *P*-value was measured by Welch's *t*-test, except for the rota-rod test. For rota-rod test, two-way ANOVA was performed to compare 2 groups, and the *p*-value in each day was analyzed by Sidak's multiple comparisons test. Each point represents data from an individual female mouse (n = 7 for control, n = 10 for Dlx1-mTOR Tg mice in (A)-(D), n = 8 for control, n = 10 for Dlx1-mTOR Tg mice in (E)-(J)).

2 Supplementary Table

Supplementary Table S1. Statistical methods and values.

Figure 2B	Two-tailed Welch's *t*-test. $t_{2.602} = 4.622$. $*p = 0.0259$.<br>Control: n = 3, Dlx1-mTOR Tg: n = 3.
Figure 2C	Two-tailed Welch's *t*-test. $t_{3.906} = 3.612$. $*p = 0.0234$.<br>Control: n = 3, Dlx1-mTOR Tg: n = 3.
Figure 2D	Two-tailed Welch's *t*-test. $t_{2.246} = 0.603$. $p = 0.6018$.<br>Control: n = 3, Dlx1-mTOR Tg: n = 3.
Figure 2E	Two-tailed Welch's *t*-test. $t_{3.432} = 6.212$. $**p = 0.0056$.<br>Control: n = 3, Dlx1-mTOR Tg: n = 3.
Figure 3C	Two-tailed Welch's *t*-test. $t_{3.467} = 3.204$. $*p = 0.0401$.<br>Control: n = 3, Dlx1-mTOR Tg: n = 4.
Figure 3D	Two-tailed Welch's *t*-test. $t_{3.903} = 3.343$. $*p = 0.0298$.<br>Control: n = 3, Dlx1-mTOR Tg: n = 4.
Figure 3E	Two-tailed Welch's *t*-test. $t_{4.198} = 3.058$. $*p = 0.0354$.<br>Control: n = 3, Dlx1-mTOR Tg: n = 4.
Figure 3F	Two-tailed Welch's *t*-test. $t_{4.759} = 6.726$. $**p = 0.0013$.<br>Control: n = 3, Dlx1-mTOR Tg: n = 4.
Figure 3G	Two-tailed Welch's *t*-test. $t_{4.972} = 1.816$. $p = 0.1295$.<br>Control: n = 3, Dlx1-mTOR Tg: n = 4.
Figure 4B	Two-tailed Welch's *t*-test. $t_{4.616} = 0.2996$. $p = 0.7775$.<br>Control: n = 3, Dlx1-mTOR Tg: n = 4.
Figure 4C	Two-tailed Welch's *t*-test. $t_{4.93} = 0.3937$. $p = 0.7103$.<br>Control: n = 3, Dlx1-mTOR Tg: n = 4.
Figure 4D	Two-tailed Welch's *t*-test. $t_{3.667} = 0.9364$. $p = 0.4066$.<br>Control: n = 3, Dlx1-mTOR Tg: n = 3.

Figure 4E	Two-tailed Welch's *t*-test. $t_{2.194}$ = 1.973. *p* = 0.1758.<br>Control: n = 3, Dlx1-mTOR Tg: n = 3.
Figure 4F	Two-tailed Welch's *t*-test. $t_{4.134}$ = 2.618. *p* = 0.0570.<br>Control: n = 3, Dlx1-mTOR Tg: n = 4.
Figure 5A	Two-tailed Welch's *t*-test. t_{12} = 2.43. **p* = 0.0317.<br>Control: n = 7, Dlx1-mTOR Tg: n = 7.
Figure 5B	Two-tailed Welch's *t*-test. $t_{11.92}$ = 2.795. **p* = 0.0163.<br>Control: n = 7, Dlx1-mTOR Tg: n = 7.
Figure 5C	Two-tailed Welch's *t*-test. $t_{11.62}$ = 0.6823. *p* = 0.5084.<br>Control: n = 7, Dlx1-mTOR Tg: n = 7.
Figure 5D	Two-tailed Welch's *t*-test. t_{12} = 0.7943. *p* = 0.4425.<br>Control: n = 7, Dlx1-mTOR Tg: n = 7.
Figure 5E	Two-tailed Welch's *t*-test. $t_{9.245}$ = 0.8345. *p* = 0.4250.<br>Control: n = 7, Dlx1-mTOR Tg: n = 7.
Figure 5F	Two-tailed Welch's *t*-test. $t_{9.534}$ = 1.1. *p* = 0.2983.<br>Control: n = 7, Dlx1-mTOR Tg: n = 7.
Figure 5G	Two-tailed Welch's *t*-test. $t_{11.12}$ = 1.159. *p* = 0.2707.<br>Control: n = 7, Dlx1-mTOR Tg: n = 7.
Figure 5H	Two-tailed Welch's *t*-test. $t_{11.7}$ = 1.116. *p* = 0.2868.<br>Control: n = 7, Dlx1-mTOR Tg: n = 7.
Figure 5I	Two-way repeated measures ANOVA.<br>Genotype: $F_{1,12}$ = 4.968, **p* = 0.0457.<br>Day: $F_{4,48}$ = 7.382, ****p* = 0.0001.<br>Interaction: $F_{4,48}$ = 2.018, *p* = 0.1069.<br>Sidak's multiple comparisons test.<br>[Day 1] Control vs. Dlx1-mTOR Tg: *p* = 0.9994.<br>[Day 2] Control vs. Dlx1-mTOR Tg: *p* = 0.9958.<br>[Day 3] Control vs. Dlx1-mTOR Tg: *p* = 0.8972.<br>[Day 4] Control vs. Dlx1-mTOR Tg: **p* = 0.0429.<br>[Day 5] Control vs. Dlx1-mTOR Tg: *p* = 0.0715.<br>Control: n = 7, Dlx1-mTOR Tg: n = 7.
Figure 5J	Two-tailed Welch's *t*-test. $t_{9.937}$ = 0.7578. *p* = 0.4661.<br>Control: n = 7, Dlx1-mTOR Tg: n = 7.

Figure 6A	Two-way repeated measures ANOVA.<br>Genotype: $F_{1,30} = 0.1141$, $p = 0.7379$.<br>Odors : $F_{8,240} = 9.601$, ****$p < 0.0001$.<br>Interaction: $F_{8,240} = 0.9316$, $p = 0.4909$.<br>Uncorrected Fisher's LSD.<br>[Control]<br>Water 1 vs. Water 2: **$p = 0.0016$<br>Water 1 vs. Water 3: **$p = 0.0012$<br>Water 3 vs. Vanilla 1: **$p = 0.0068$<br>Vanilla 1 vs. Vanilla 2: *$p = 0.0450$<br>Vanilla 1 vs. Vanilla 3: *$p = 0.0116$<br>Vanilla 3 vs. Almond 1: ***$p = 0.0007$<br>Almond 1 vs. Almond 2: **$p = 0.0010$<br>Almond 1 vs. Almond 3: ***$p = 0.0004$<br>[Dlx1-mTOR Tg]<br>Water 1 vs. Water 2: *$p =0.0322$<br>Water 1 vs. Water 3: *$p = 0.0176$<br>Water 3 vs. Vanilla 1: ****$p < 0.0001$<br>Vanilla 1 vs. Vanilla 2: ****$p < 0.0001$<br>Vanilla 1 vs. Vanilla 3: ****$p < 0.0001$<br>Vanilla 3 vs. Almond 1: **$p = 0.0011$<br>Almond 1 vs. Almond 2: $p = 0.1251$<br>Almond 1 vs. Almond 3: **$p = 0.0038$<br>Control: n = 15, Dlx1-mTOR Tg: n = 17.
Figure 6B	Two-tailed Welch's t-test. $t_{30} = 0.3296$. $p = 0.7440$.<br>Control: n = 15, Dlx1-mTOR Tg: n = 17.
Figure 6C	Two-tailed Welch's t-test. $t_{28.37} = 0.8089$. $p = 0.4253$.<br>Control: n = 15, Dlx1-mTOR Tg: n = 17.
Figure 6E	Two-way repeated measures ANOVA.<br>Genotype: $F_{1,29} = 0.03687$, $p = 0.8491$.<br>Odors: $F_{1,29} = 16.3$, ***$p = 0.0004$.<br>Interaction: $F_{1,29} = 2.634$, $p = 0.1154$.<br>Uncorrected Fisher's LSD.<br>[Control] Peanuts vs. Fox urine: ***$p = 0.0006$<br>[Dlx1-mTOR Tg] Peanuts vs. Fox urine: $p = 0.0829$<br>Control: n = 14, Dlx1-mTOR Tg: n = 17.
Figure 7B	Two-way Ordinary measures ANOVA.<br>Genotype: $F_{1,8} = 37.79$, ***$p = 0.0003$.<br>Odors: $F_{1,8} = 35.62$, ***$p = 0.0003$.<br>Interaction: $F_{1,8} = 27.52$, ***$p = 0.0008$.<br>Sidak's multiple comparisons test.<br>Water (Control) vs. Odors (Control): ***$p = 0.0002$<br>Water (Control) vs. Water (Dlx1-mTOR Tg): $p = 0.9971$<br>Odors (Control) vs. Odors (Dlx1-mTOR Tg): ***$p = 0.0003$<br>Water (Dlx1-mTOR Tg) vs. Odors (Dlx1-mTOR Tg): $p = 0.9907$<br>Water (Control): n = 3, Odors (Control): n = 3<br>Water (Dlx1-mTOR Tg): n = 3, Odors (Dlx1-mTOR Tg): n = 3.

Supplementary Figure 1B	Two-tailed Welch's *t*-test. $t_{3.81}$ = 2.216. p = 0.0943.<br>Control: n = 3, Dlx1-mTOR Tg: n = 3.
Supplementary Figure 1C	Two-tailed Welch's *t*-test. $t_{3.007}$ = 1.489. p = 0.2331.<br>Control: n = 3, Dlx1-mTOR Tg: n = 3.
Supplementary Figure 1D	Two-tailed Welch's *t*-test. $t_{3.53}$ = 0.4877. p = 0.6545.<br>Control: n = 3, Dlx1-mTOR Tg: n = 3.
Supplementary Figure 1E	Two-tailed Welch's *t*-test. $t_{3.583}$ = 0.4768. p = 0.6611.<br>Control: n = 3, Dlx1-mTOR Tg: n = 3.
Supplementary Figure 2A	Two-tailed Welch's *t*-test. $t_{14.46}$ = 3.809. **p = 0.0018.<br>Control: n = 7, Dlx1-mTOR Tg: n = 10.
Supplementary Figure 2B	Two-tailed Welch's *t*-test. $t_{14.65}$ = 1.894. p = 0.0782.<br>Control: n = 7, Dlx1-mTOR Tg: n = 10.
Supplementary Figure 2C	Two-tailed Welch's *t*-test. $t_{11.51}$ = 0.556. p = 0.5889.<br>Control: n = 7, Dlx1-mTOR Tg: n = 10.
Supplementary Figure 2D	Two-tailed Welch's *t*-test. $t_{13.82}$ = 0.9532. p = 0.3569.<br>Control: n = 7, Dlx1-mTOR Tg: n = 10.
Supplementary Figure 2E	Two-tailed Welch's *t*-test. $t_{13.95}$ = 0.3382. p = 0.7402.<br>Control: n = 8, Dlx1-mTOR Tg: n = 10.
Supplementary Figure 2F	Two-tailed Welch's *t*-test. $t_{13.54}$ = 0.7689. p = 0.4552.<br>Control: n = 8, Dlx1-mTOR Tg: n = 10.
Supplementary Figure 2G	Two-tailed Welch's *t*-test. $t_{15.14}$ = 0.4653. p = 0.6483.<br>Control: n = 8, Dlx1-mTOR Tg: n = 10.
Supplementary Figure 2H	Two-tailed Welch's *t*-test. $t_{15.8}$ = 1.43. p = 0.1722.<br>Control: n = 8, Dlx1-mTOR Tg: n = 10.
Supplementary Figure 2I	Two-way repeated measures ANOVA.<br>Genotype: $F_{1,16}$ = 6.435, *p = 0.0220.<br>Day: $F_{4,64}$ = 20.85, ****p < 0.0001.<br>Interaction: $F_{4,64}$ = 1.175, p = 0.3303.<br>Sidak's multiple comparisons test.<br>[Day 1] Control vs. Dlx1-mTOR Tg: p = 0.7650.<br>[Day 2] Control vs. Dlx1-mTOR Tg: p = 0.2657.<br>[Day 3] Control vs. Dlx1-mTOR Tg: p = 0.5193.<br>[Day 4] Control vs. Dlx1-mTOR Tg: *p = 0.0308.<br>[Day 5] Control vs. Dlx1-mTOR Tg: *p = 0.0388.<br>Control: n = 8, Dlx1-mTOR Tg: n = 10.
Supplementary Figure 2J	Two-tailed Welch's *t*-test. t_{16} = 2.437. *p = 0.0268.<br>Control: n = 8, Dlx1-mTOR Tg: n = 10.

* $p < 0.05$, ** $p < 0.01$, *** $p < 0.001$, **** $p < 0.0001$
